# Nitrogen limitation causes a seismic shift in redox state and phosphorylation of proteins implicated in carbon flux and lipidome remodeling in *Rhodotorula toruloides*

**DOI:** 10.1186/s13068-025-02657-y

**Published:** 2025-07-21

**Authors:** Austin Gluth, Jeffrey J. Czajka, Xiaolu Li, Kent J. Bloodsworth, Josie G. Eder, Jennifer E. Kyle, Rosalie K. Chu, Bin Yang, Wei-Jun Qian, Pavlo Bohutskyi, Tong Zhang

**Affiliations:** 1 Biological Sciences Division, Pacific Northwest National Laboratory, Richland, WA USA; 2 Department of Biological Systems Engineering, Washington State University, Richland, WA USA; 3 Energy and Environment Directorate, Pacific Northwest National Laboratory, Richland, WA USA; 4 Agile BioFoundry, US Department of Energy, Emeryville, CA USA

## Abstract

**Background:**

Oleaginous yeast are prodigious producers of oleochemicals, offering alternative and secure sources for applications in foodstuff, skincare, biofuels, and bioplastics. Nitrogen starvation is the primary strategy used to induce oil accumulation in oleaginous yeast as part of a global stress response. While research has demonstrated that post-translational modifications (PTMs), including phosphorylation and protein cysteine thiol oxidation (redox PTMs), are involved in signaling pathways that regulate stress responses in metazoa and algae, their role in oleaginous yeast remain understudied and unexplored.

**Results:**

Towards linking the yeast oleaginous phenotype to protein function, we integrated lipidomics, redox proteomics, and phosphoproteomics to investigate* Rhodotorula toruloides* under nitrogen-rich and starved conditions over time. Our lipidomics results unearthed interactions involving sphingolipids and cardiolipins with ER stress and mitophagy. Our redox and phosphoproteomics data highlighted the roles of the AMPK, TOR, and calcium signaling pathways in regulation of lipogenesis, autophagy, and oxidative stress response. As a first, we also demonstrated that lipogenic enzymes including fatty acid synthase are modified as a consequence of shifts in cellular redox states due to nutrient availability.

**Conclusions:**

We conclude that lipid accumulation is largely a consequence of carbon rerouting and autophagy governed by changes to PTMs, and not increases in the abundance of enzymes involved in central carbon metabolism and fatty acid biosynthesis. Our systems-level approach sets the stage for acquiring multidimensional data sets for protein structural modeling and predicting the functional relevance of PTMs using Artificial Intelligence/Machine Learning (AI/ML). Coupled to those bioinformatics approaches, the putative PTM switches that we delineate will enable advanced metabolic engineering strategies to decouple lipid accumulation from nitrogen limitation.

**Supplementary Information:**

The online version contains supplementary material available at 10.1186/s13068-025-02657-y.

## Background

Low-cost organic wastes from agricultural and forestry industries provide renewable avenues for production of biofuels, bioproducts, and biomaterials [1]. Oleaginous yeast are exceptional at converting these complex carbon sources into lipids, proteins, and other platform chemicals [2–7]. The carotenoid-producer* Rhodotorula toruloides* is an oft-studied chassis for oil production because of its native capacity to utilize biomass-derived disaccharides, hexoses, pentoses, organic acids, and aromatics as well as its high tolerance to inhibitory byproducts of lignocellulose pretreatment [8–14]. Under carbon-replete conditions and nitrogen, sulfur, or phosphate limitation,* R. toruloides* and other oleaginous yeast will accumulate neutral lipids, primarily in the form of triacylglycerides (TGs) [15–17]. Nitrogen limitation is the main strategy to trigger lipid overproduction, although this intervention presents a clear dilemma regarding resource availability for antioxidants, proteins, and reducing cofactors.

Nutrient limitation disrupts the homeostasis between reducing and oxidizing (redox) reactions underlying metabolism and leads to a response reminiscent of oxidative stress [18–20]. In model non-oleaginous yeast, antioxidant proteins and the synthesis of the small molecule antioxidant glutathione are upregulated in response to nitrogen starvation [21–23]. In recent years, an appreciation for the physiological importance of pro-oxidant reactive oxygen species (ROS) and antioxidants has translated to research involving lipogenesis in oleaginous microorganisms—though primarily in microalgae and bacteria [24–30]. There are, however, key studies that indicate the significance of redox balance for lipid production in oleaginous yeast. For instance, proteomics investigations of* R. toruloides* report a relative increase in antioxidants such as glutathione peroxidase during nitrogen limitation [31, 32]. A link between redox homeostasis, fitness and lipid metabolism was investigated in metabolic engineering studies of carotenoid-overproducing* R. toruloides* and the model oleaginous yeast* Yarrowia lipolytica* [33, 34]. Liu et al. (2023) showed that higher ROS levels are in part due to fatty acid β-oxidation, respiration, and iron metabolism, which may contribute to higher production of antioxidant carotenoids in a mutant strain; however, a direct comparison between nitrogen-rich and nitrogen-limited conditions was not evaluated [33]. Xu et al. (2017) demonstrated that overexpression of antioxidants improved both fitness and lipid production, yet fundamental questions pertaining to metabolic regulation were not addressed [34].

During nutrient limitation, ROS can function as secondary messengers to propagate stress-induced signals and regulate protein function [20, 24, 35]. ROS such as hydrogen peroxide (H_2_O_2_) and superoxide (O_2_^−^) are byproducts of aerobic respiration, fatty acid β-oxidation, disulfide bond formation, nitrogen scavenging, and other metabolic processes [36–38]. These pro-oxidants can oxidize protein cysteine thiols leading to a spectrum of reversible modifications (redox PTMs) including sulfenylation (-SOH), glutathionylation (-SSG), and disulfide bonds (S–S) [39, 40]. The regulatory role of redox PTMs has been studied in metazoa, cyanobacteria, bacteria, and model yeasts [41–44]; however, there are no investigations in oleaginous yeast despite nitrogen requirements for antioxidant and reducing cofactor synthesis and the importance of lipids for redox and energy homeostasis. The role of other regulatory PTMs especially protein phosphorylation in cell signaling and metabolism is well-established for ascomycete model yeast; however, the phosphoproteomes of oleaginous yeast have rarely been studied. In oleaginous yeast, the first phosphoproteomics study was performed in the ascomycete* Y. lipolytica*, revealing a change in the phosphorylation levels for many key lipid metabolism proteins including ATP–citrate lyase and acetyl-CoA carboxylase [45].

**Fig. 1:**
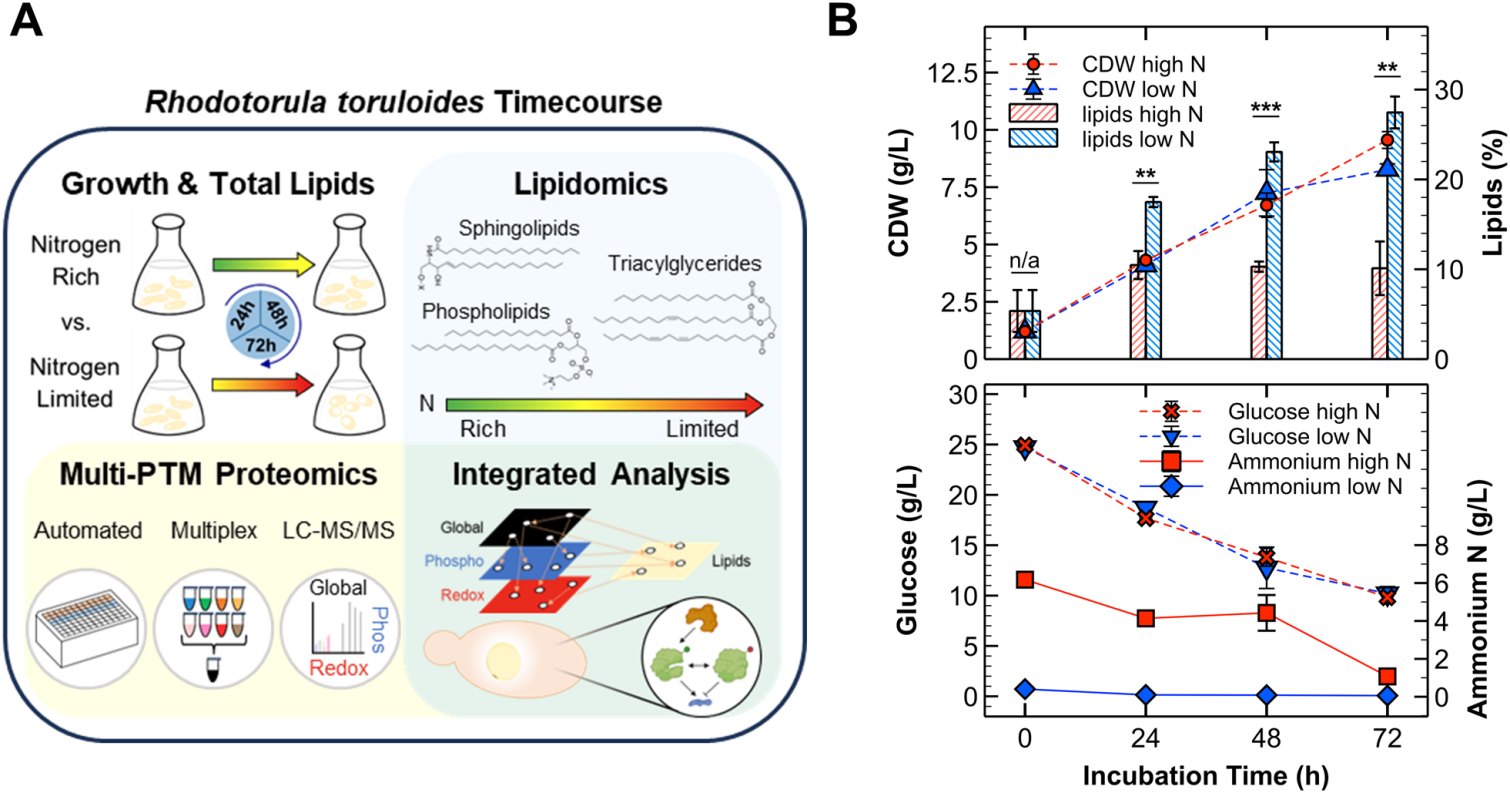
Growth and lipid kinetics for the multi-omics experimental approach. **A** Experimental approach for integrated multi-omics analysis. Briefly,* R. toruloides* was cultivated in nitrogen-rich (C:N of 5:1) and limited (C:N of 90:1) conditions with sampling at 24, 48, and 72 h. For growth and lipid kinetics, a 0 h timepoint was included to analyze media and the inoculum. Lipidomics and multiplexed quantitative proteomics/PTMomics analyses were conducted to attain an integrative perspective of protein regulation, cellular PTM states, and metabolism. **B** Line plots demonstrating biomass production (CDWs; cell dry weights) and substrate consumption (glucose and ammonium). The overlayed bar chart shows lipid contents for the different conditions. Error bars represent one standard deviation from biological triplicates (except for 0 h in which technical replicates were evaluated). For the bar chart, significance levels from two-sample *t*-tests are included: ** = *p-*value ≤ 0.01, *** = *p-v*alue ≤ 0.001

We hypothesize that nitrogen limitation causes major shifts in thiol oxidation and phosphorylation of multiple protein classes, notably regulatory proteins in the highly conserved AMP kinase (AMPK) and TOR pathways, and metabolic enzymes tasked with re-routing carbon flux for lipidome remodeling [46–49]. To establish a connection between protein regulation and the oleaginous phenotype of* R. toruloides*, we employed a multi-omics approach that integrates multi-PTM proteomics profiling and lipidomics analyses (Fig. 1A). We used our semi-automated proteomics approach to quantify changes in protein abundance (global proteomics), cysteine thiol oxidation (redox proteomics), and phosphorylation (phosphoproteomics) using multiplexed sample inputs [50–52]. This workflow not only unveiled candidate redox-regulated proteins but also facilitated a deeper investigation of multi-PTM interplay in oleaginous yeast. These candidates included enzymes central to fatty acid biosynthesis, such as fatty acid synthase, ATP–citrate lyase, and acetyl-CoA carboxylase. Modifications to TOR, AMPK, calcium, and mitogen-activated protein kinase (MAPK) signaling pathways coincided with downregulation of nitrogen-containing phospholipids and sphingolipids. Because these signaling pathways are central to nutrient-sensing, resource-monitoring, and stress response, we conclude that lipid accumulation is driven by autophagic recycling processes that steadily pump excess carbon to TGs, while nitrogen is scavenged to maintain anabolic requirements for cell survival and stress response—including antioxidants. These results reveal novel PTM-based regulatory mechanisms and putative redox switches with potential for expanding synthetic biology tools to improve fitness and lipid production of oleaginous yeasts.

## Results and discussion

### Growth and lipid production kinetics for timecourse multi-omic collection

A single timecourse experiment of *R. toruloides* cultivated on 25 g/L glucose was conducted to compare nitrogen-rich (C:N of 5:1) and limited (C:N of 90:1) conditions. We analyzed growth and lipid production kinetics, lipidome compositions, and differentially expressed proteomes/PTMomes at 24, 48, and 72 h after inoculation (Fig. 1A). Total biomass production, measured as cell dry weights (CDWs), remained similar between nitrogen-rich and limited conditions up to 48 h, with continued growth observed only in nitrogen-rich cultures. Differences in lipid titers were observed as early as 24 h, consistent with reports in other yeast (Fig. 1B) [45]. Comparing the substrate yields for total biomass vs. lipids (Additional File 1, Table S1), it is clear that carbon flux differed for the two conditions in spite of the similar CDWs. The yeast cells reached an average lipid content of 27.5% under nitrogen starvation compared to ~ 10% at all timepoints under high nitrogen conditions (Fig. 1B). The rate of glucose utilization was similar for both conditions, with ~ 10 g/L glucose remaining at 72 h (Additional File 1, Table S1). In the low nitrogen condition, most of the ammonium was consumed within 24 h and 1 g/L remained after 72 h in the high nitrogen condition, thereby providing a range of nitrogen concentrations for lipidomics and proteomics analyses (Fig. 1B).

Previous studies with *R. toruloides* reported a CDW of ~ 9 g/L at 72 h under similar conditions (C:N of ~ 100), with another recent study reporting a ~ 2 g/L difference in CDWs between C:N ratios of 20 and 80 (using ammonium nitrate) [9, 53]. Our lipid titers aligned with these findings, although the particular strain, starting OD_600_, nitrogen source, and aeration (shaker rpm) impacted the outcomes [9, 53–55]. For instance, the rate of glucose utilization was the same for both conditions with ~ 10 g/L glucose remaining at the end of our experiment. This has also been observed by others using an inorganic nitrogen source, with similar growth and lipid production parameters reported (Additional File 1, Table S1) [53, 56, 57].

### Lipidomics analysis reveals dynamic lipid profiles under varied nitrogen availability

**Fig. 2:**
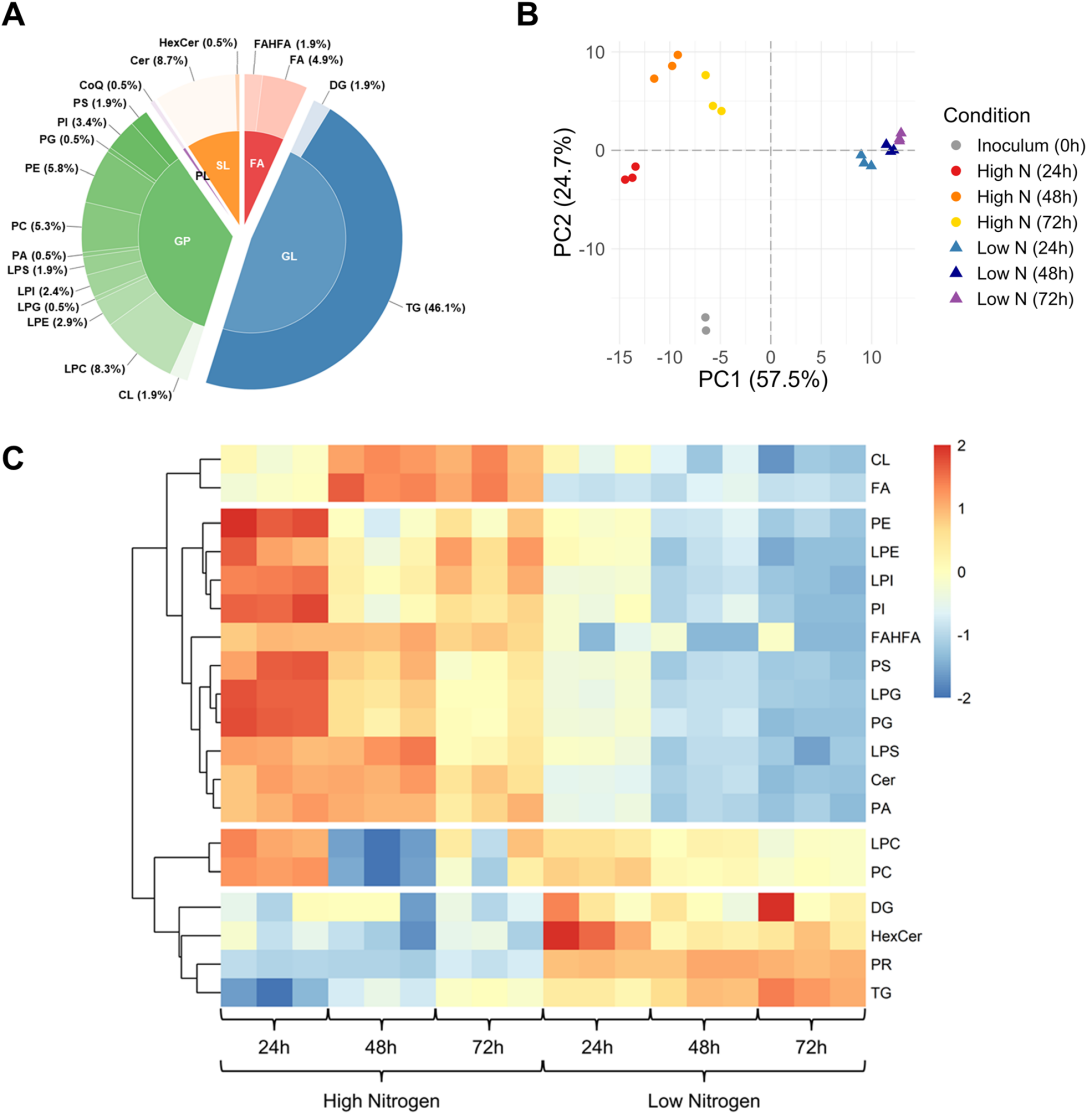
Lipidome dynamics according to nitrogen availability. **A** Nested pie chart presenting the distribution of unique lipid IDs in the major classes: glycerophospholipids (GC), glycerolipids (GL), fatty acyls (FA), sphingolipids (SL), and prenol lipids (PR). Lipid subclass abbreviations are as follows: coenzyme Q (CoQ), ceramide (Cer), cardiolipin (CL), prenol lipid (PR), triacylglyceride (TG), diacylglyceride (DG), fatty acid (FA), fatty acid ester of hydroxy fatty acid (FAHFA), glycosphingolipid (HexCer), phosphatidylcholine (PC), phosphatidylethanolamine (PE), phosphatidylglycerol (PG), phosphatidylinositol (PI), phosphatidylserine (PS), phosphatidic acid (PA), and the corresponding lyso-derivatives (e.g., LPC). **B** Principal component analysis of normalized lipidomics data. The 0 h condition (inoculum) was evaluated in technical replicates, but all other conditions were investigated using biological triplicates. **C** Heatmap showing summed peak heights for lipids in the observed subclasses. The data was transposed and scaled to a mean of zero and standard deviation of 1 prior to visualization (see the legend on the far right)

Lipids are integral to cellular membranes, cell signaling, redox homeostasis, and survival/death [58, 59]. The lipidomes of* R. toruloides* and related species have been characterized under a variety of conditions: different lignocellulose-derived carbon sources, nitrogen-rich and limited media, and rich undefined media over time [33, 54, 60–62]. However, these previous studies focused on storage lipids and often did not capture signaling pathways involving lipids or the role of lipids in mitophagy and autophagy, which are key processes for resource turnover and modulating lipid droplet formation [63–65, 65]. We observed 206 unique lipid species, with 172 glycerophospholipids and glycerolipids (~ 83% of the observed lipids), 19 sphingolipids, 14 fatty acyls, and one prenol lipid (Fig. 2A). Notably, many cardiolipins, fatty acid ester of hydroxy fatty acids (FAHFAs), and the signaling lipid phosphatidylinositol (PI) 18:0/18:0 [66] were not observed in the low nitrogen conditions (Additional File 2). Cardiolipins are predominantly localized in the mitochondrial inner membrane, where they stabilize proteins of the electron transport chain [67]. Though there are few studies in yeast, FAHFA is another subclass of bioactive lipids implicated in the regulation of glucose and lipid metabolism [68–70]. Following normalization, we conducted principal component analysis (PCA) to probe the effect of nitrogen availability and culture time on lipidome variation (Fig. 2B). High and low nitrogen samples clustered separately along PC1, while cultivation time within each nitrogen condition contributed to PC2 (e.g., the separation between 24 and 72 h samples). These results are echoed in a heatmap showing the summed peak heights for the different lipid subclasses: interestingly, phospholipids and TGs demonstrate a decreasing and increasing trends over time in both conditions, respectively (Fig. 2C).

To unravel significant lipid signatures among the culture conditions, we performed differential expression and orthogonal partial least squares–discriminant analyses (OPLS–DA) (Additional File 1, Figs. S1–2). Time-matched differential analysis showed an upregulation of sphingolipids and fatty acyl lipids in the high nitrogen conditions compared to the low nitrogen conditions, with the significance level dropping over time (Additional File 1, Fig. S1, top row). Timepoint comparisons within the same nitrogen availability group showed a clear upregulation of phospholipids and downregulation of glycerolipids (Additional File 1, Fig. S1, bottom row). This is consistent with the influence of inorganic nitrogen availability (especially per CDW) on lipid metabolism: as more nitrogen is utilized, glycerolipids are upregulated, while phospholipids are downregulated [54]. The significance levels and degree of this effect are lower for cells that have already exhausted exogenous nitrogen at 24 h. To reduce the complexity imposed by combinatorial comparisons, differential expression was also conducted using nitrogen and time coefficients in a linear model (Additional File 1, Fig. S1B). This approach was employed for OPLS–DA, too; both analyses demonstrated the dominant impact of the nitrogen grouping variable and pinpointed ceramides, cardiolipins, phosphatidylethanolamines (PEs), and TGs as discriminating lipids (Additional File 1, Figs. S1–2).

Enrichment analyses aided in prioritizing lipid species that warrant additional consideration with respect to nitrogen availability. Lipid species enrichment analyzes (LSEA) supported trends observed in differential expression analysis and OPLS–DA and provided additional insights about lipid unsaturation. For the pairwise comparisons between high and low nitrogen conditions, ceramides, phosphatidylcholines (PCs), PEs, and TGs were significantly enriched (Additional File 1, Fig. S3A). Ceramides are primarily found in the plasma membrane and play key roles in membrane structure and signaling pathways [58, 71]. PC and PE phospholipids contribute substantially to membrane fluidity and curvature [58, 72] and were downregulated in the high nitrogen timecourse comparisons, while TGs were upregulated as nitrogen became limiting.

Acyl chain unsaturation also affects membrane architecture and, consequently, the function of membrane proteins [73–75]. According to LSEA, polyunsaturated fatty acyls were enriched in low nitrogen conditions (Additional File 1, Fig. S3B). Desaturases use NADH to convert saturated fatty acids into unsaturated forms [74], which exemplifies the connection between lipid metabolism and redox balance. It follows that omega-9 fatty acid synthesis was a pathway enriched in our results. Other enriched pathways include sphingolipid metabolism, fatty acid biosynthesis and transport, as well as glycerophospholipid catabolism (Additional File 1, Fig. S4). The latter is related to LPC hydrolysis, which may provide fatty acids for synthesis of TG storage lipids. It is important to note here that many lipids are not annotated well or do not even have a corresponding metabolic pathway or biological process annotation, which is a limitation of lipidomics pathway enrichment analysis in this non-model organism. Nevertheless, anticipated shifts in TGs and phospholipids, combined with unexplored changes in ceramides and FAHFAs, provide several pathways for integrating proteomics results.

### Nitrogen limitation causes a systematic shift in protein thiol oxidation and phosphorylation

**Fig. 3:**
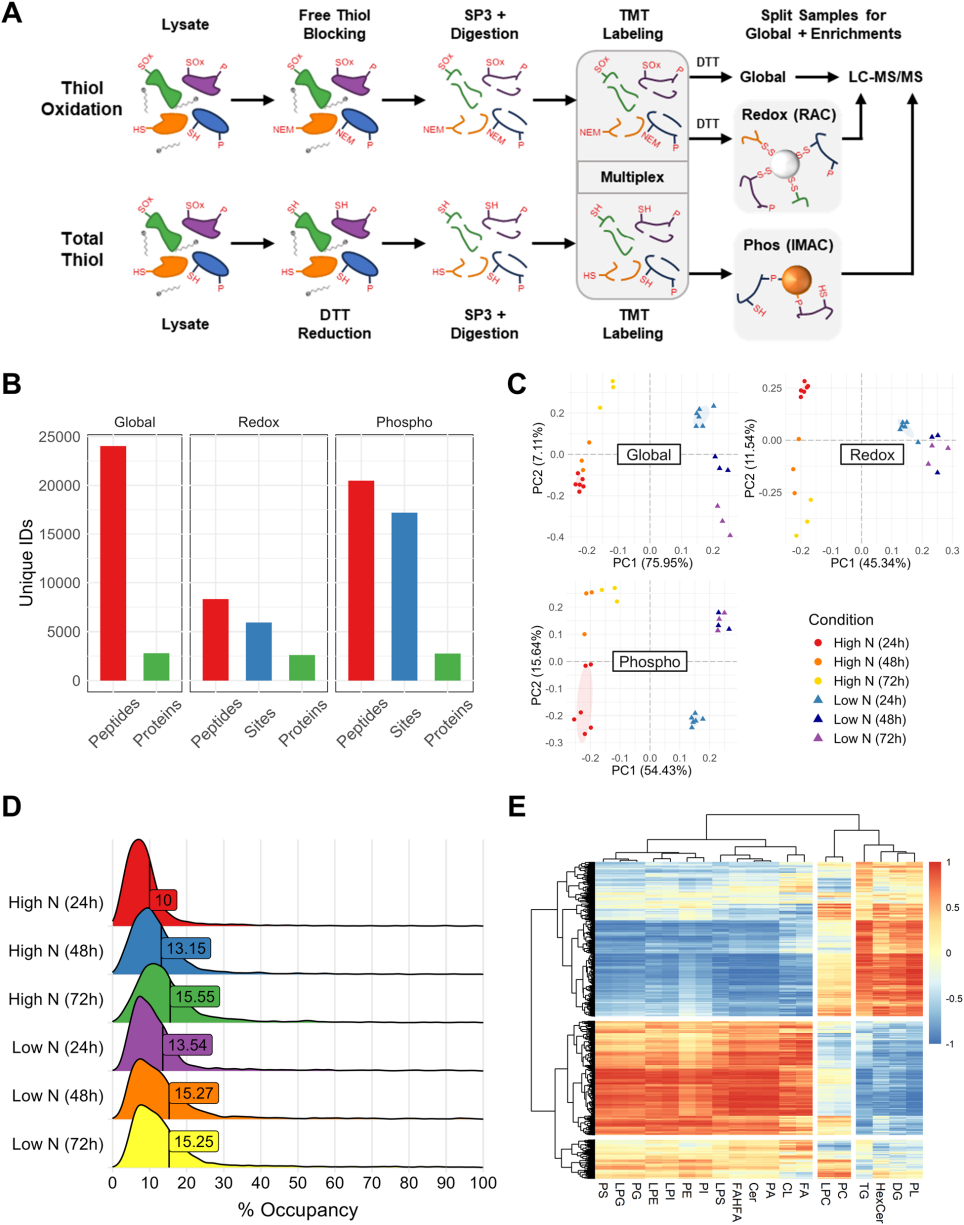
Protein thiol oxidation and phosphorylation patterns are distinguished by nitrogen availability and cultivation time. **A** Workflow for multiplexed quantification of global protein abundance, cysteine thiol oxidation, and phosphorylation. During homogenization, protein free thiols in “Thiol Oxidation” samples were blocked with NEM, whereas NEM was omitted for “Total Thiol” samples. Automated single-pot, solid-phase-enhanced sample preparation (SP3) was then used for sample cleanup, followed by on-bead digestion and TMT labeling. After desalting, samples were split for protein abundance analysis and enrichment of PTMs. Reversibly oxidized cysteine thiols (represented by “-SOx”) were reduced and enriched by resin-assisted capture (RAC), while phosphorylated peptides were captured using immobilized metal affinity chromatography (IMAC). **B** Faceted bar chart presenting unique peptide, PTM site, and protein identifications for each sample type. **C** PCA plots with ellipses delineating the 95% confidence intervals for the 24 h replicates. **D** Distributions of percent cysteine thiol oxidation (the “% Occupancy” of thiol oxidation abundances/total thiol abundances). The population mean value is labeled on each distribution. **E** Heatmap showing Pearson correlation between the summed lipid subclass intensities and normalized thiol oxidation abundances

Though there is a wealth of PTM proteomics data in model yeast [76–78], simultaneous quantification of multiple PTM types in oleaginous yeast has not been reported. As a first, we employed our new multi-PTM approach [52] to probe the multifaceted effects of nitrogen limitation on protein phosphorylation and thiol oxidation (Fig. 3A). To enrich oxidized cysteine residues for relative quantification, two sets of samples (derived from the same cultures) were processed in parallel: one set termed “thiol oxidation” in which reduced cysteine free thiols are blocked with* N*-ethylmaleimide (NEM) and another termed “total thiol” in which all cysteines are reduced using DTT. Automated single-pot, solid-phase-enhanced sample preparation (SP3) was then employed for sample cleanup and peptide digestion. The resulting peptides were isobarically labeled with TMT, which facilitated the pooling and subsequent splitting of samples for enrichment of PTM-containing peptides (i.e., thiol oxidation and phosphorylation) and analysis of global protein abundances.

In total, we identified 2,804 unique proteins in the global samples, 5,926 cysteine sites in the redox proteomics samples, and 17,195 sites in the phosphoproteomics samples with a 1% peptide-level FDR (Fig. 3B). This allowed us to investigate the relative abundances of PTMs on > 2,000 proteins. For all three omics data types (global, phosphorylation, and redox), PCA showed a clear separation based on the nitrogen condition along PC1 and cultivation time along PC2 (Fig. 3C). Within the low-nitrogen samples, 24 h samples were clearly separated from those at 48 h and 72 h (which clustered together) in redox and phosphoproteomics data, suggesting earlier regulatory events. These trends are recapitulated in clustered heatmaps of Pearson correlations, which additionally support PTM dynamics according to nitrogen availability (Additional File 1, Fig. S5A). Results of differential expression analyses are presented in Additional File 1, Fig. S5B and demonstrate the sensitivity of the approach for uncovering significant changes in protein abundance and PTMs (Additional File 3). Hundreds of proteins and PTM sites were significantly up- and downregulated at each timepoint (adjusted *p*-value cutoff of 0.05). Using a further absolute log_2_FC cutoff of 0.8, we noted more significant cysteine residues with lower oxidation levels (291 downregulated vs. 187 upregulated) at 72 h, which was also observed in the phosphorylation data for the presented comparisons (e.g., 411 downregulated vs. 249 upregulated at 72 h).

To investigate the role of redox regulation under nitrogen-limiting conditions, the percentage of oxidized thiols was calculated using the ratio of “Thiol Oxidation” and “Total Thiol” samples (Fig. 3D and Additional File 4). We found a mean % of 10–15% thiol oxidation for the six sample groups, agreeing well with the only two other yeast redox proteomics studies [78, 79]. We observed a 20–35% increase in mean % thiol oxidation between high and low nitrogen conditions for the 24 h and 48 h timepoints, with a 1.5-fold increase in thiol oxidation between 24 and 72 h. A lack in difference at 72 h may be due to diminishing nutrient availability, cell aging, culture density (and oxygen availability), and/or changes to medium pH. As an additional systems-level overview of cysteine oxidation and lipid metabolism, we correlated the average abundances of the oxidized cysteine sites with the summed averages of the lipid subclass data (Fig. 3E). We noted that TGs and DGs as well as the prenol lipid coenzyme Q9 (CoQ9) cluster together, whereas phospholipids, sphingolipids, and cardiolipins cluster in a separate parental tree. As with carotenoids, CoQ9 serves to protect the cell against ROS [80] and is upregulated in the low nitrogen condition. We performed KEGG pathway enrichment analysis on cysteine sites with Pearson correlation coefficients > 0.80 for TG and DG and absolute log_2_FCs ≥ 0.8 using a general linear model according to nitrogen availability (see Materials and methods section; Additional File 1, Fig. S6). Amino acid and nitrogen metabolism were enriched in addition to proteasomal and ribosomal processes. Importantly, fatty acid metabolism was upregulated—the specific enriched genes in this pathway were fatty acid synthase FAS1 and ERG10, which is involved in first step of ergosterol synthesis. The full set of KEGG pathway enrichment results for global, redox, and phosphoproteomics data is presented in Additional File 1 and Fig. S7. These results directed our focus in the following subsections on nitrogen recycling, central carbon and lipid metabolism, and signaling pathways using protein annotations from JGI [81] and eggNOG [82].

### Nitrogen metabolism and autophagy are differentially regulated at protein and PTM levels

**Fig. 4:**
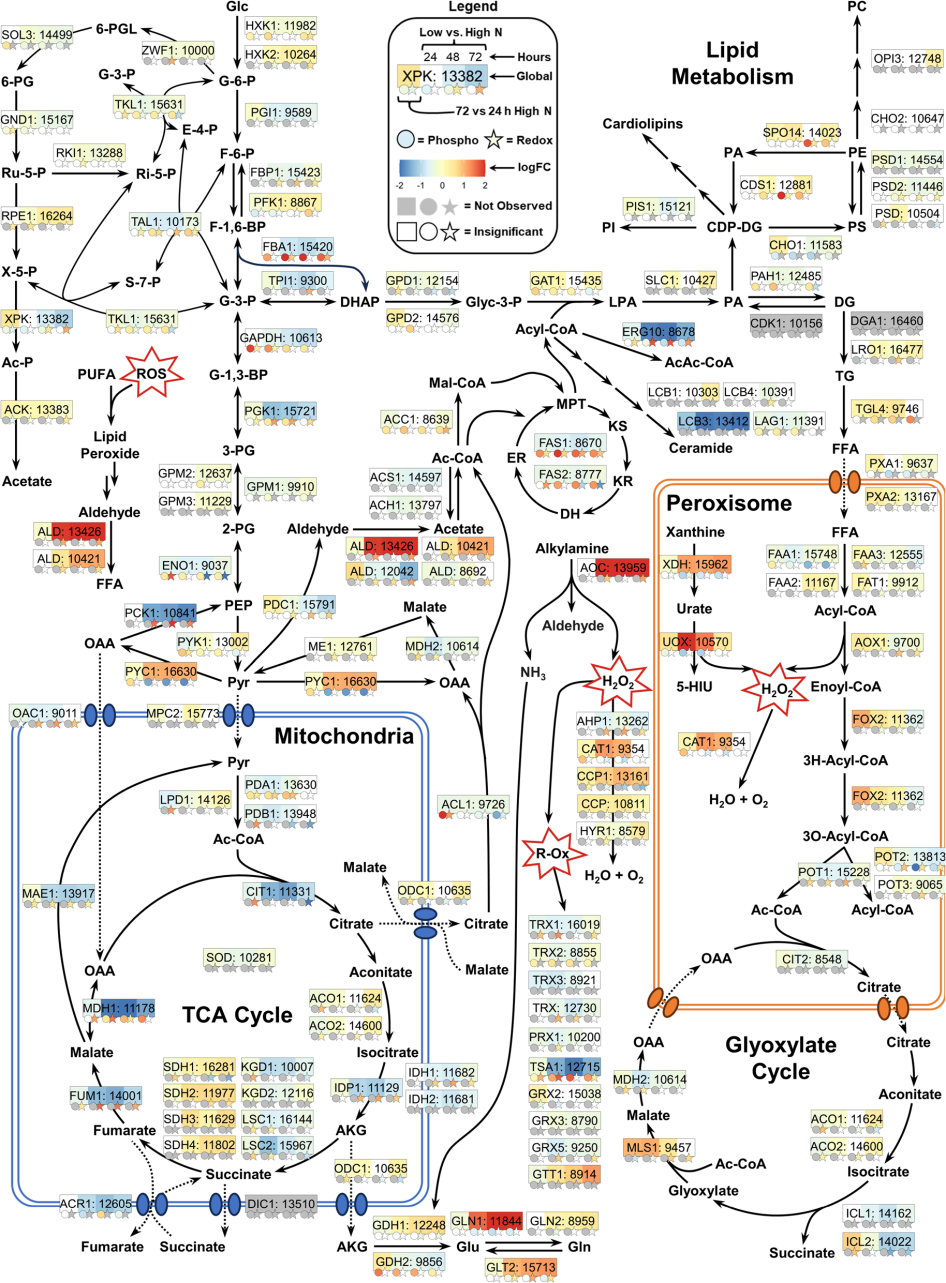
Summary of multi-PTM proteomics results for central carbon metabolism and lipogenesis. Metabolic pathways constructed based on annotations aggregated from JGI [81], eggNOG [82], as well as BLASTp results against S. cerevisiae S288C [87] (http://www.yeastgenome.org/) and* R. toruloides* NP11 [83]. Labels include common gene nomenclature abbreviations and the corresponding JGI accession ID. Details for gene and metabolite abbreviations are provided in Additional File 6 along with several additional annotations for desaturases, elongases, lipooxygenases, and enzymes involved in carotenoid and sterol synthesis. The gene label box is split into four sections describing relative protein abundances for four comparisons. The first is 72 vs. 24 h of high nitrogen, and the remaining three are low vs. high nitrogen at 24, 48, and 72 h. Circle (phosphorylation) and star (thiol oxidation) labels for each of the four comparisons provide a snapshot of PTM data for one particular residue (details for all observed residues found in Additional File 6). Grey and white labels define proteins and/or PTM sites that were not observed or did not exhibit significant changes, respectively. Multiple arrows denote multiple reaction steps, while dotted arrows indicate transport. Enzyme annotations for cardiolipin metabolism are not included, because they were not observed

Scavenging for available nitrogenous resources is a hallmark response to nitrogen limitation [31, 83]. Under these conditions, nitrogen catabolite depression induces the expression of transporters, autophagic enzymes, and pathways for utilizing alternative nitrogen sources, such as urea, urate, ammonia, nitrate/nitrite, amides, etc. As expected, multiple permeases and transporters were upregulated with some exhibiting large changes in PTMs (e.g., log_2_FC 5.33 for Rt_8962 S20 at 48 h; Additional File 5). This was also the case for enzymes of central nitrogen metabolism, which include glutamate dehydrogenase (GDH), glutamine synthase (GLN), and glutamate synthase (GLT) (Fig. 4). For instance, GDH2 phosphorylation at residue T24 or S26 was upregulated (log_2_FC 1.11) during nitrogen limitation at 48 h. A couple other examples include oxidation of C1774 in the FAD-binding dihydroprymidine dehydrogenase domain II (IPR028261) of GLT1 (log2FC 1.10 at 48 h) as well as a relatively more reduced state of C251 in the conserved ATP-binding signature (IPR027302) and conserved glycine rich site (IPR027303) of GLN1 (log_2_FC − 1.77 at 48 h). In addition to enrichment of nitrogen metabolism, expression of genes involved in urea degradation was upregulated, which are implicated in the urea cycle module of arginine metabolism (Additional File 1, Fig. S7).

Numerous peptidases, proteases, ATPases, and Rab GTPases involved in vacuolar protein degradation and autophagy were induced during nitrogen limitation to promote resource recycling. These include previously reported PRB1 and PEP4, the latter of which activates vacuolar hydrolases to enhance degradation of macromolecules [83]. In our time-matched comparisons using minimal medium, we observed upregulation (log_2_FC ≥ 0.8) of vacuolar-type ATPases (VMA3, VMA4, and VMA8), a target t-SNARE for vesicle transport (PEP12), and autophagy-related protein 27 (ATG27) which was also observed in another proteomics study [84]. As a transmembrane protein, ATG27 is required for ATG9 cycling to target and sequester membranes for pre-autophagosome formation [85]. Cysteine residues C27 and C188 of ATG27 had significantly higher oxidation states (log_2_FC 1.10 and 1.15 at 48 h, respectively). Analysis using InterProScan and alignment with* S. cerevisiae* ATG27 could not confirm whether these oxidized cysteine residues form disulfide bonds [86, 87]. Mitophagy is also important for lipid homeostasis [63, 88]: we observed downregulation of the GTPase DNM1 and upregulation of several phosphorylated residues, which may modulate mitochondrial organization and fission (Additional File 5) [89, 90].

Autophagy has been observed in parallel with increased ROS generation during nitrogen limitation [20], evidencing a relationship between nitrogen scavenging, organelle stability, and resource availability for redox homeostasis [91–93]. Hydrogen peroxide is produced to a high extent as a byproduct of reactions catalyzed by oxidases that are involved in amino acid, purine, and fatty acid metabolism. An amine oxidase (EC 1.4.3.21; Rt_13959) was among the highest upregulated proteins in our results (log_2_FC 2.23 at 48 h) and those of another study [31]. This enzyme deaminates short-chain primary amines to produce ammonia as well as reactive aldehyde and hydrogen peroxide byproducts [94]. C440 of amine oxidase displayed increased oxidation (log_2_FC 1.15 at 72 h) and is directly adjacent to signature for amiloride-induced enzyme inhibition (PR00766) [86]. Interestingly, a positive correlation between microalgal lipid accumulation and amine oxidase expression under nitrogen limitation has been reported [95]. Purine degradation is another ROS-generating route for recycling nitrogen and was upregulated in our KEGG pathway enrichment results (Additional File 1, Fig. S7).* R. toruloides* possesses a xanthine dehydrogenase (XDH) that facilitates purine catabolism via allantoin to ultimately release ammonia. As with* Y. lipolytica*, XDH was upregulated in* R. toruloides* during nitrogen starvation (log_2_FC 1.24 at 72 h) [96]. The urate produced from XDH-mediated purine degradation is converted to 5-hydroxyisourate by a urate oxidase, which was upregulated 2.32-fold at 24 h.

### Carbon flux to lipogenesis is regulated in part by PTMs—not drastic changes to enzyme expression

To visualize protein and PTM abundance patterns, we mapped our proteomics data onto a summary of metabolic pathways including glycolysis, PPP, TCA, the glyoxylate cycle, and lipid synthesis (Fig. 4). For many metabolic enzymes, we found that nitrogen limitation leads to little or no change to protein abundances, but significant changes at PTM levels. In glycolysis, fructose 1,6-bisphosphate aldolase (FBA1) catalyzes the conversion of fructose 1,6-bisphosphate to glyceraldehyde-3-phosphate (G3P) and dihydroxyacetone–phosphate (DHAP). We observed downregulation of FBA1 protein abundance (log_2_FC–0.92 at 48 h) and significant upregulation of phosphorylation at S262 at all three timepoints in the nitrogen-limited conditions (e.g., log_2_FC 2.07 at 24 h). FBA1^S262^ is putatively conserved in* S. cerevisiae* according to ClustalOmega [97] alignment, and FBA1 can be phosphorylated by cyclin B/cyclin-dependent kinase (CDK1)* in vitro* [98], tying together regulation of carbon metabolism and cell cycling (results of which are discussed in the following subsection). Triose phosphate isomerase (TPI1) interchangeably converts G3P to DHAP and has a conserved redox-sensitive C126 [78, 79, 99], which displayed increased cysteine oxidation (log_2_FC 1.03 at 48 h). Several additional enzymes in glycolysis—particularly around the glycerol metabolic branch point—had upregulated thiol oxidation and/or phosphorylation events: glyceraldehyde 3-phosphate dehydrogenase (GAPDH), phosphoglycerate kinase (PGK1), and enolase (ENO1) (Fig. 4; Additional File 6). In addition, we observed increased oxidation of fructose-1,6-bisphosphatase (FBP1; log_2_FC 0.92 at 48 h for C171), which converts fructose-1,6-bisphosphate to fructose 6-phosphate in gluconeogenesis. FBP1^C171^ is located in the active site and may be regulated by ROS [100, 101]. These results suggest that nitrogen limitation leads to a cellular state partially reminiscent of oxidative stress induced by hydrogen peroxide [78], heavy metals [99], and antioxidant deletions [79].

Enzymes in the TCA cycle as well as mitochondrial carboxylic acid transporters were generally downregulated (e.g., citrate synthase CIT1 with log2FC − 1.57 at 48 h) during nitrogen limitation. In contrast, pyruvate carboxylase (PYC1), a cytosolic enzyme that catalyzes the carboxylation of pyruvate to oxaloacetate (OAA), was upregulated in all time-matched comparisons (e.g., log_2_FC 1.07 at 48 h). OAA is subsequently converted to malate by a cytoplasmic malate dehydrogenase (MDH2) that cycles back to pyruvate by the action of NADP + -dependent malic enzyme (ME1), producing NADPH for lipid synthesis [102]. Another key enzyme for lipid synthesis is ATP–citrate lyase (ACL1), which converts mitochondria-derived citrate to OAA and acetyl-CoA. Phosphoregulation of ACL1 has been reported previously [103], and several residues in our results displayed differential phosphorylation levels: S706 (log2FC − 2.57), S499 (log_2_FC 1.02), and S512;T513 (log_2_FC − 1.49) for the 72 h comparison.

In addition to ME1, several other enzymes may provide NADPH for fatty acid synthesis, antioxidants, and other anabolic processes in* R. toruloides* [31, 102]. These include glucose-6-phosphate dehydrogenase (ZWF1), NADP^+^ -dependent isocitrate dehydrogenase (IDP1), NADP ^+^ -dependent glyceraldehyde-3-phosphate dehydrogenase, and NADP ^+ ^-dependent aldehyde dehydrogenases (Fig. 4). Interestingly, ZWF1 showed higher oxidation at C241/247 (log_2_FC 1.05 at 24 h), which are predicted to bear oxidative modification including S-sulfenylation and S-sulfhydration (false positive rate between 2.68% and 4.62%, respectively) according to pCysMod [104]. The putative regulatory role of cysteine oxidation in modulating flux through the PPP has been studied in plants and cyanobacteria [105–108].

With acetyl-CoA and NADPH, fatty acid biosynthesis proceeds towards a portfolio of lipid species. The first committed step involves acetyl-CoA carboxylase (ACC1) that carboxylates acetyl-CoA to form malonyl-CoA. We observed phosphorylation of S1047 (log_2_FC 0.87 at 24 h) and relative dephosphorylation of S1159 at all timepoints (e.g., log_2_FC − 1.38 at 72 h). In S*. cerevisiae, *this residue is conserved and is phosphorylated by the master regulator AMP kinase (signal pathways detailed in the next subsection) to downregulate ACC1 activity [109–111]. We also observed increased cysteine oxidation on ACC1^C1315^ (log_2_FC 1.02 at 72 h and 1.29 for low nitrogen 72 h vs. 24 h), which is part of the central domain located N-terminally to the catalytic domain (IPR013537) [86]. Following malonyl-CoA synthesis, fatty acid synthase (FAS1/2) catalyzes a series of condensation, reduction, and elongation steps [72] to produce long-chain acyl-CoAs. Several thiols exhibited differential oxidation levels in FAS1: C825 and C1106 in the central domain of the beta subunit (IPR013565; log_2_FC 1.08 at 48 h and log2FC − 1.42 at 72 h, respectively), C509 and C567 in the acyl transferase domain (IPR001227; log_2_FC 1.04 at 48 h and log_2_FC − 1.34 at 72 h, respectively), as well as C1163 (log_2_C − 1.62 at 72 h) [86]. Adding to the PTM mosaic, multiple residues at the N-terminus of FAS1 had increased phosphorylation (Additional File 6).

Most of the synthesized fatty acids are incorporated into TG and phospholipids. Our data showed that differences in protein abundances were negligible for proteins involved in lipid metabolism, but we observed several instances of increased phosphorylation that may promote TG accumulation (Fig. 4). Phosphatidic acid (PA) is the key intermediate in phospholipid and TG synthesis. It is dephosphorylated to DG by phosphatidate phosphatase (PAH1; aka lipin): phosphorylation of PAH1^S1005^ was upregulated (log_2_FC 0.90 at 24 h). Although it is unclear if this specific residue is involved in regulation, phosphoregulation of PAH1 in* S. cerevisiae* is conferred by the TOR signaling pathway [112–114]. Biosynthesis of membrane phospholipids (e.g., PI, PS, and PC) involves the attachment of a head group to PA, which is catalyzed by phosphatidate cytidylyltransferase (CDS1). In addition, PE can be converted to PA and ethanolamine (a nitrogen scavenging strategy) by phospholipase D (SPO14). Both CDS1 and SOP14 showed residues with increased phosphorylation levels (Additional File 6). We also noted that ERG10 and LCB3, a serine palmitoyltransferase involved in the first step of sphingolipid biosynthesis, were downregulated (e.g., log_2_FC − 1.47 and − 3.43 at 72 h, respectively). The latter corroborates our lipidomics results (Fig. 2C) and raises questions about sphingolipid regulation of autophagy and growth arrest during nitrogen-limited lipid accumulation. This is because ceramides are known to bind several proteins including the aspartate protease cathepsin D (log_2_FC 1.64 at 48 h; Additional File 6). Finally, we observed increased phosphorylation of a multifunctional lipase (TGL4), which liberates fatty acids from lipids for subsequent β-oxidation [115] and indicates the involvement of intricate signaling pathways controlling carbon flux during nitrogen starvation.

### PTM-mediated signaling networks orchestrate nitrogen scavenging, suppress protein synthesis, control lipid production, and arrest cell cycling

**Fig. 5:**
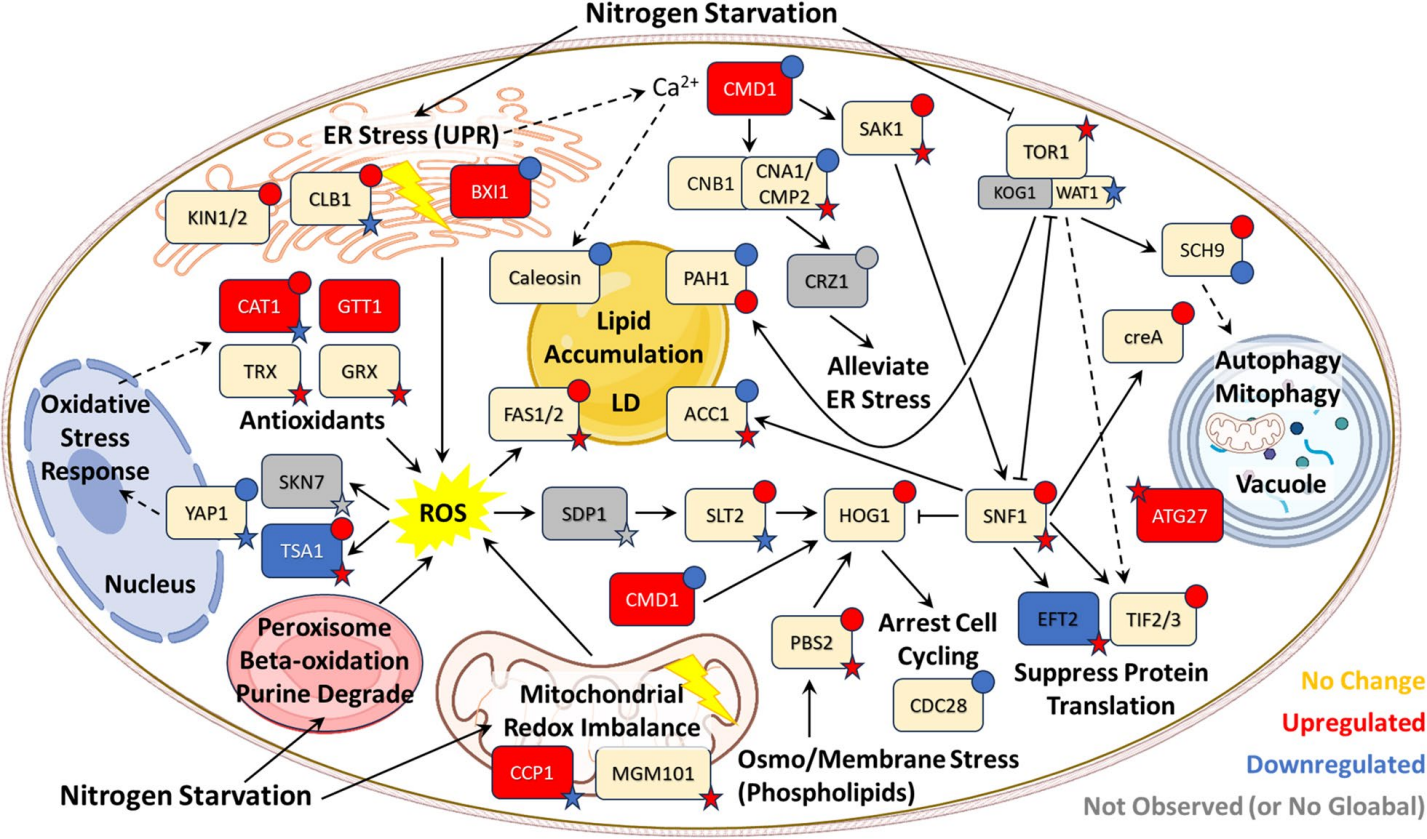
Significant proteome changes in signaling pathways that regulate stress response and metabolism according to nitrogen availability. Labels include common gene nomenclature abbreviations referenced herein and defined in Additional File 7. Circle (phosphorylation) and star (thiol oxidation) labels provide a snapshot of PTM data for one residue. In many cases (e.g., PAH1), multiple residues exhibited significant changes, but for the sake of simplicity, proteins with significant changes in at least one of the comparisons are summarized in the pathway portion of this figure. Grey labels define proteins that were not observed. Dotted arrows represent multiple steps

Nitrogen limitation regulates carbon flux, lipid accumulation, and stress response by modulating several key signaling pathways including the TOR, AMPK, calcium signaling, and MAPK pathways (Fig. 5 and Additional File 1, Fig. S9). While TOR signaling senses the availability of nitrogenous compounds and regulates autophagy, SNF1 (mammalian ortholog AMPK) acts as a sensor of energy status working in tandem with TOR. Calcium signaling plays a pivotal role in orchestrating interactions between the ER, lipid droplet, and mitochondria [72, 116–118]. The interplay between TOR, AMPK, and ER stress-induced calcium signaling ensures cellular survival during nitrogen limitation through the lipid accumulation phenotype and autophagy [119, 120] while the MAPK pathway integrates stress signals to arrest cell cycling. Our multi-PTM proteomics results allowed us to explore protein expression, phosphorylation, and thiol oxidation patterns in these regulatory networks. These results underlined the complex interplay among these pathways in regulating autophagy, protein translation, and lipid production among other salient processes in response to nutrient availability (Fig. 5) [46, 121]. These laboriously curated annotations of signaling pathway proteins provide a vast collection of candidates to study the functional roles of PTMs in unconventional oleaginous yeast.

TORC1 is a master regulatory complex that is active under optimal growth conditions, thereby positively regulating anabolic processes such as protein synthesis and repressing catabolic process like autophagy [122]. TORC1 is comprised of the catalytic subunit TOR1 as well as KOG1 and WAT1, which are responsible for recruiting target proteins and stabilizing interactions with TOR1, respectively [123, 124]. In this work, nitrogen limitation caused no change in TOR1 abundance but increased oxidation at TOR1^C275^ (log_2_FC 0.81 at 24 h and 0.96 for high nitrogen 72 h vs. 24 h), a residue in the HEAT repeats that mediates protein–protein interactions (including with KOG1) [125–127]. The AGC kinase SCH9 is a major substrate of TORC1 in yeast [128–130]. In* S. cerevisiae*, TORC1 phosphorylates SCH9 at several residues in response to amino acid availability [131, 132]. We observed increased phosphorylation on SCH9^S750^ (log_2_FC 1.03 and 0.94 at 24 and 48 h, respectively; Additional File 7). Along with the serine/threonine kinase GCN2, the transcriptional activator GCN4, and the AMP kinase SNF1, the TORC1 and SCH9 axis is involved in regulating translation [128, 133–136]. We observed significant changes in phosphorylation of translation initiation factor eIF4α/β (TIF2 or TIF3 based on BLASTp results) at several residues including T386 (log_2_FC 2.38-fold at 48 h) and T355 (log_2_FC − 1.57 at 48 h). Additionally, an array of phosphorylation events was observed for upstream regulators of TORC1, including the RAG family of small GTPases (GTR1 and GTR2 in yeast) and the SEA complexes, which facilitate amino acid sensing and signaling events (Additional File 7) [122, 137].

The ER is the site of lipid droplet formation and central to calcium storage and homeostasis (Fig. 5) [118]. Nitrogen starvation causes ER stress, during which Ca^2+^ is released to trigger stress responses. We observed upregulation of calmodulin (CMD1; log_2_FC 1.31 at 24 h), a calcium-binding receptor that mediates stress-activated signaling pathways. During nitrogen starvation, CMD1 activates the Ca^2+^/calmodulin-dependent protein kinase SAK1 (mammalian ortholog CaMKKβ), which in turn activates SNF1 via phosphorylation. Interestingly, , and deletion of SAK1 increases lipid accumulation in* Y. lipolytica* [138–141]. SAK1 is a regulatory hub for integrating diverse signaling pathways including TOR, SNF1, calcium, MAPK, and cAMP (Additional File 1, Fig. S8) [140, 142]. SAK1 displayed differential phosphorylation and thiol oxidation on several residues including T333 (log_2_FC 1.75 at 72 h) and C145 (log_2_FC 1.16 at 48 h), though the functions are unclear based on alignment and domain analyses [86, 97]. Phosphorylation of SNF1 was slightly upregulated at T345 and T347 (log_2_FC 0.80 and 0.75, respectively), which are residues that are not conserved in* S. cerevisiae* SNF1 [97]. In a similar mechanism for protein synthesis control as TORC1, SNF1 inhibits protein synthesis by promoting phosphorylation of eukaryotic elongation factor-2 (EFT2) [143]. Even though we did not observe EFT2 in our phosphoproteomics results, we did note increased thiol oxidation on EFT2^C416^ and EFT2^C561^ (log_2_FCs of 2.03 and 1.08, respectively, at 24 h) and decreased oxidation on EFT2^C200^ (log_2_FC − 1.16 at 72 h). EFT2^C200^ is part of a GTP-binding signature (PR00315) [86], and cysteine thiol oxidation can reversibly inhibit elongation factor EF-Tu in cyanobacteria [144]. Other targets of SNF1 include MIG1 (creA) which regulates carbon catabolite repression (and displayed C-terminal hyperphosphorylation [145, 146] in our results) and HOG1 (an ortholog of human p38α, discussed below), supporting the broad involvement of SNF1 in amino acid metabolism, translation, and fatty acid β-oxidation [96].

In addition to SAK1, CMD1 activates calcineurin (a calcium/calmodulin-dependent phosphatase) and calmodulin-regulated kinases [147]. Yeast calcineurin is encoded by two catalytic subunits (CNA1 and CMP2) and a regulatory subunit CNB1 [148]. In CMP2, we noted oxidation of C186 (log_2_FC 1.05 at 72 h), which is immersed in the metallophosphatase domain (cd07416) [86]. Numerous phosphorylation events were also observed (Additional File 7) including downregulation of S622 (log2FC − 2.01 at 48 h). Intriguingly, calcineurin is activated by mitochondrial ROS overproduction and dysregulation of lipid metabolism [149, 150]. Furthermore, it activates the ER stress response transcription factor CRZ1/TCN1, which affects the expression of genes related to sphingolipid and sterol synthesis [148, 151]. Additional markers of ER stress displaying differential regulation of protein abundance and/or PTMs include KIN2, a serine/threonine kinase that regulates IRE1-mediated unfolded protein response [152]; CLB1, a B-type cyclin involved in cell cycle progression [153]; CDC28, a cyclin-dependent kinase [147, 154]; and BXI1, a member of the BAX inhibitor family that controls apoptosis in response to calcium homeostasis [155].

In further support of the connection between calcium signaling and modulation of cell cycling in response to stress, Kim et al. showed that fungal mitogen-activated protein kinase HOG1 binds calmodulin [156]. Moreover, SNF1 negatively regulates HOG1 during ER stress [157]. HOG1 has primarily been studied for its role in cellular adaptation to high osmolarity [158]. The osmosensing machinery upstream of HOG1 includes the membrane proteins SLN1 and SHO1, which associate with lipid rafts that are rich in sphingolipids and sterols [159, 160]. Interestingly, Tanigawa et al. inhibited sphingolipid synthesis and noted activation of the MAPK signaling pathway [159]. HOG1 deletion in the fungal pathogen* Candida albicans* induces lipid accumulation during osmotic stress [161]. We observed an increase in TGs and a decrease in sphingolipids (Fig. 2C) during nitrogen limitation, which paralleled upregulation of HOG1 phosphorylation at residues Y173 (log_2_FC 1.12 at 72 h) and Y171 (log_2_FC 1.01 for low nitrogen 72 h vs. 24 h). InterProScan analysis revealed that Y173 lies within* R. toruloides* HOG1’s catalytic domain active site (cd07856) [86]. We also observed a decrease in cardiolipins which is consistent with mitophagy and may be linked to MAPK signaling transduction. Even during nitrogen limitation, the cell still has to regulate cellular envelope stability and turgor pressure to survive and does so through protein kinase C (PKC1) and the serine/threonine MAP kinase SLT2 [162]. PKC1 and SLT2 also regulate mitophagy [163] and had significant changes in phosphorylation (Additional File 7). Importantly, HOG1 regulates expression of catalase expression [164], and the upstream MAP kinase phosphatase SDP1 has been shown to be redox-sensitive [165, 166].

### Antioxidant expression and oxidative stress signaling are perturbed by nitrogen availability

Nitrogen starvation activates oxidative stress response [18, 22], yet this connection has not been studied in oleaginous yeast. Nitrogen starvation can trigger the accumulation of ROS via ER stress, fatty acid β-oxidation in peroxisome, nitrogen scavenging, and autophagy-related mitochondrial redox imbalance (Additional File 1, Fig. S10). As part of the antioxidant defense response, we observed upregulation of peroxisomal/cytoplasmic catalase (CAT1), two mitochondrial cytochrome-c peroxidase (CCPs), glutathione S-transferase (GTT1), and aldehyde dehydrogenases (ALDs) (Fig. 4 and Additional File 6). Upregulation of CCPs is consistent with mitochondrial instability during nitrogen starvation given that these enzymes function as both mitochondrial hydrogen peroxide sensors and antioxidants [167, 168]. Furthermore, we were amazed to see oxidation of ribosome-associated thioredoxin peroxidase (TSA1) at C48 and C170 (log_2_FC 1.66 and 1.27 at 24 h, respectively). Both TSA1^C48^ and TSA1^C170^ are conserved in the* S. cerevisiae* strain S288C homolog, which react with hydrogen peroxide and form intermolecular disulfide bonds with another TSA1 or the transcriptional activator YAP1 [169–171]. YAP1 moderates oxidative stress response and is itself regulated by intramolecular disulfide bonds [172, 173]. We observed only one cysteine-containing peptide for YAP1. This peptide had a decrease in oxidation at C480 (log_2_FC − 2.0 at 72 h), which aligns with C620 of ScYAP1 and may be involved in nuclear localization [172–175]. Interestingly, we also observed upregulated phosphorylation at S130;S136 (log_2_FC 1.23 at 72 h)—immediately adjacent to the bZIP DNA binding domain (IPR004827) [86].

## Conclusions

In this study, we employed a recently developed semi-automated multi-PTM proteomics approach to study protein thiol oxidation and phosphorylation in the oleaginous yeast *R. toruloides* under nitrogen limitation—a bioprocessing condition critical for triggering oil production yet conducive to oxidative stress [20, 34]. Going one step further, we systematically studied the lipidomes from the same timecourse experiment to delineate putative regulatory connections among redox homeostasis, autophagy, central carbon metabolism, and lipogenesis.

In our multi-omics analysis, downregulation of sphingolipids and cardiolipins was concomitant with upregulation of nitrogen scavenging and autophagic/mitophagic processes. Based on our results and prior knowledge about signaling pathways and the phosphatidic acid regulatory node, we conclude that TG accumulation is driven in part by an increased input of acyl-CoA. This input stems from phospholipid and sphingolipid catabolism as well as acetyl-/propionyl-CoA from nitrogen scavenging. Despite our extensive analysis of metabolic and signaling pathways, prominent challenges remain. First and foremost, proteomics is a powerful tool for discovery and validation as we exemplify; however, wading through the deluge of protein and PTM results complicates the selection of strain engineering targets. This is compounded by the fact that most functional studies have been conducted in* S. cerevisiae*, a non-oleaginous ascomycete yeast, and to a lesser extent* Y. lipolytica*, an oleaginous ascomycete yeast. In contrast,* R. toruloides* is an oleaginous carotenoid-producing basidiomycete yeast, and though many biological processes are conserved, there are likely important evolutionary differences [46]. As a last point, even with model organisms, omics methods are challenged by the total coverage of macromolecules that can be identified [176]. For instance, we did not identify key regulators of nitrogen catabolite repression and autophagy, such as URE2 and ATG4, both of which possess cysteine residues engaged in antioxidant-related functions [93, 177].

While the complexities of omics analyses present significant challenges in understanding non-model basidiomycete yeast, emerging technologies and integrative approaches offer promising avenues for overcoming these obstacles. For instance, chemoselective probe-based approaches would enable direct investigation of specific types of redox PTMs, rather than a general overview of cysteine thiol oxidation [178, 179]. Lipids themselves undergo a plethora of oxidative modifications including peroxidation and enzyme-mediated oxygenation, potentially transforming them into critical signaling molecules involved in stress response [180, 181]. We observed upregulation of a putative linoleate 8R-lipoxygenase (log_2_FC 0.93 at 48 h; Additional File 1, Fig. S10 and Additional File 6); however, as with other fungi, the functional roles of oxylipins are unclear [182]. Importantly, future studies will be required to probe the functional consequences of cysteine thiol oxidation and phosphorylation events on the yeast oleaginous phenotype. High-throughput evaluation of differentially modified candidates will be facilitated by techniques such as protein structural modeling [183] and genetic engineering approaches for site-specific point mutations—or even permanent installations of PTM mimetics [184]. Our multi-PTM multi-omics results not only set the stage for optimizing stress response and lipid production in* R. toruloides* but also underscore the emerging significance of PTMs in synthetic biology research.

## Materials and methods

### Strain, media compositions, and growth conditions

*Rhodotorula toruloides* strain IFO0880 (now called NBRC 0880) [185] was maintained on YPD (20 g/L glucose, 20 g/L peptone, and 10 g/L yeast extract) agar plates. A single large colony was inoculated into 200 mL YPD in 1 L flat bottom flasks and incubated at 30 °C and 180 rpm shaking for 24 h. The starter culture was pelleted via centrifugation at 6,500 rpm for 5 min and washed twice with the low nitrogen experimental medium. To set up the experimental culture, 50 mL of high or low nitrogen medium in 250 mL flat bottom flasks were inoculated in triplicate to a starting OD_600_ of 0.5. The remaining yeast cells from the starter cultures were pooled, flash-frozen in liquid nitrogen, and stored for a 0 h lipidomics timepoint. The two experimental media differed only by the concentration of NH_4_Cl: 7.1 g/L (C:N of 5:1) for the high nitrogen condition and 0.4 g/L (C:N of 90:1) for the low nitrogen condition. Both contained 25 g/L glucose, 1.7 g/L YNB (without amino acids and (NH_4_)_2_SO_4_; BD Difco), 25 mM Na_2_HPO_4_, and 150 mM KH_2_PO_4_ with a final pH of 5.6. The media were filter-sterilized using 0.22 µm PES filters (CellTreat). C:N ratios and medium composition were chosen according to previous research [54, 186].

Experimental cultures were incubated at 30 °C and 160 rpm to avoid aeration-induced stress and were harvested at 24, 48, and 72 h, based on previous proteomics investigations [31, 83]. 20 mL of culture was partitioned into tared conical tubes for cell dry weights and total gravimetric lipids analyses, while another 20 mL was collected for proteomics. Samples were centrifuged, and 10 mL of supernatant was transferred to a new tube for metabolite analyses. Cell pellets were washed twice with buffer containing 25 mM Na_2_HPO_4_ and 150 mM KH_2_PO_4_ (pH 5.6). During the last wash, a total of 2 OD units was removed from each culture (in technical duplicates) for proteomics sample processing. All samples were flash-frozen in liquid nitrogen and stored at − 80 °C.

### Cell dry weight and gravimetric total lipids analyses

Frozen cell pellets were dried in a Labconco lyophilizer overnight and were then weighed on an analytical balance to record cell dry weights. The resulting pellets were crushed in a mortar and pestle, and 50 mg of ground sample was transferred to 2 mL bead beater tubes containing 0.5 mm glass beads (Omni International). A modified Bligh and Dyer (1959) approach was used for total lipids extractions [187, 188]. 600 µL of 4:3 v/v methanol:water was added to each tube, and bead beating was performed with a Bead Ruptor Elite (Omni International) using 6 repeats of 30 s beating cycles (6.0 m/s) with 2 min ice incubations after each cycle. A needle (0.45 mm × 10 mm; BD) was used to carefully poke a hole in the bottom of the bead beater tubes, which were inserted into 2 mL vials (Corning) and centrifuged at 2500 × g for 3 min at RT. The lysate was then transferred to 15 mL chloroform-compatible conical tubes (Olympus Plastics), and an additional 400 µL of 4:3 v/v methanol:water was added to wash the bead beater tubes. 3 mL of 343:1000:1540 v/v/v water:chloroform:methanol solution was added to the combined lysate to attain an approximate ratio of 0.8:1:2 water:chloroform:methanol. Each tube was vortexed for 1 min then mixed for 1 h at room temperature (RT) via 130 rpm shaking with intermittent vortexing every 15 min. Afterwards, 1 mL of chloroform and 1 mL of water was added to each sample to attain a final ratio of 1.8:2:2 water:chloroform:methanol. Each sample was vortexed for 1 min and then centrifuged at 7,100 × * g* for 10 min to promote phase separation. The bottom chloroform layer was transferred to pre-tared 2 mL Sorenson tubes using glass Pasteur pipettes. Finally, the chloroform was evaporated in a vacuum concentrator (SpeedVac), and the tubes were weighed on an analytical balance.

### Determining extracellular glucose and ammonium concentrations

Glucose concentrations in the fermentation broth were quantified by high performance liquid chromatography (HPLC). 1 mL of supernatant was filtered through a 0.2 µm nylon filter then transferred into a glass vial. 10 µL of sample was loaded onto an Aminex HPX-87H column at a flow rate of 0.6 mL/min using 4 mM sulfuric acid as a mobile phase and a column temperature of 65 °C. A refractive index detector was used for analysis.

Ammonium concentrations were quantified using the indophenol blue assay [189]. Supernatant samples from biological triplicates were processed in technical duplicates and were diluted 10–1000-fold to a final volume of 10 mL using ultrapure water. After which, the following were added: 0.4 mL of reagent (10% w/v phenol solution in ethanol), 0.4 mL catalyst (0.5% w/v nitroprusside in water) and 1 mL oxidizing solution (a 4:1 v/v mixture of 20% w/v trisodium citrate containing 1% w/v sodium hydroxide solution and a 5% v/v sodium hypochlorite solution). Samples were incubated in the dark at 37 °C with 160 rpm shaking for 2 h. Absorbance was read at 630 nm and concentrations were calculated using a standard curve of 0.125–5 g/L ammonium chloride.

### LC–MS/MS for lipidomics samples

Total lipid extracts (TLEs) in this study were analyzed by reverse-phase LC–MS/MS using a Thermo Scientific Vanquish Flex UHPLC system (Thermo Scientific) coupled with a Fusion Lumos mass spectrometer (Thermo Scientific) as described previously [60, 190]. TLEs were resuspended in 500 µL of 2:1 v/v chloroform:methanol at a final concentration of 0.066 µg/µL and transferred to Waters vials for − 20 °C storage. Samples were dried and reconstituted in 500 µL of 9:1 v/v methanol:chloroform prior to injecting 10 µl onto a Waters column (CSH 3.0 mm × 150 mm × 1.7 µm particle size) maintained at 42 ºC. Lipid species were separated using a 34 min gradient elution at a 250 µL/min flow rate. Mobile phases A and B consisted of 40:60 v/v acetonitrile:H_2_O containing 10 mM ammonium acetate, and 10:90 v/v acetonitrile:isopropanol also containing 10 mM ammonium acetate, respectively. Each TLE was analyzed in both positive and negative ion modes in separate runs. Lipids were fragmented using both higher energy collision dissociation and collision-induced dissociation following a precursor scan of m/z 120–1,800 at a mass resolution of 120 k.

### Lipidomics data processing and analysis

Raw mass spectrometry data was processed using MS-DIAL [191], and confident identifications were made by examining the diagnostic ion fragments and associated chain fragments in tandem mass spectra. First, MS-DIAL’s Lipidblast databases were used to match spectra to identify lipids. Then, the mass error, extracted ion chromatogram (XIC), and retention time were manually examined, after which peak apex intensity values were recorded for subsequent statistical analysis.

Bioinformatics analysis of the lipidomics data sets was conducted in RStudio [192] using the lipidr package [193]. Missing values were treated as “0” and converted to “1” prior to log-transformation and probabilistic quotient normalization [193, 194]. Missing values were then converted to NAs, which were not filtered prior to limma [195] differential expression analysis given that mass spectra were manually evaluated, and a particular lipid must be observed in one of the samples. Rank-based LSEA was performed with lipidr using log_2_ foldchanges [193]. MetaboAnalyst 6.0 [196] was used for over-representation analysis of enriched metabolite sets (MSEA–ORA; pathway enrichments) that were acquired by filtering differential expression results using an absolute log_2_FC cutoff of 4 with an adjusted* p*-value ≤ 0.05. The RaMP database [197] was chosen using metabolite sets with at least 2 entries; the list of all lipid species identified in this study (Additional File 2) was used as the reference set. Overall, 53 of the 206 lipid species could not be assigned by MetaboAnalyst. Figures were created and/or edited using the ggplot2 and pheatmap packages [198, 199].

### Cell lysis and automated proteomics sample processing using SP3 magnetic beads

Sample preparation for multi-PTM profiling was performed according to our established method [52]. Cell pellets were thawed in 500 μL of lysis solution containing 250 mM MES (pH 6.0), 5% SDS, and 1% Triton X-100 with or without 100 mM NEM. The pellets were resuspended by pipetting and homogenized via bead beating as described above. An additional 100 μL of the lysis solution was used to wash residual lysate off the beads. The supernatant was transferred to 1.5 mL tubes, and protein concentrations were measured by the BCA assay.

Automation using SP3 magnetic beads was performed on KingFisher Flex (Thermo Scientific) with slight modifications to the approach employed by Leutert et al. [51]. These included an increase in sample volumes to accommodate higher protein loadings as well as an additional step in which the magnetic comb returns to the sample/binding plate to collect residual beads to transfer to the first wash plate. Information regarding the SP3 magnetic beads (Cytiva) and pre-washing steps is included in the Additional File 1, Supplementary Methods.

In a deep-well plate (sample/binding plate), lysate containing ~ 500 μg protein was transferred, and sample volumes were adjusted to 500 μL using ultrapure water. Then, 500 μL of absolute ethanol was added to each well. The bead plate was prepared prior to lysis and contained 200 μL of 25 μg/μL washed SP3 beads. Three wash plates containing 800 μL of 80% ethanol were prepared last using a multichannel pipette. The comb, bead, sample/binding, and wash plates were loaded onto KingFisher, and the program was started. During the washing steps, the digestion plate was prepared with each well containing 500 μL of digestion mixture (1:100 trypsin and 1:100 LysC in 50 mM HEPES buffer, pH 7.7). After washing, the digestion plate was loaded, and beads were transferred by KingFisher. Digestion was performed offline on an incubator (3 h at 37 °C with 500 rpm shaking). Near the end of the digestion step, 500 μL of 100 mM HEPES (pH 8.0) with 5% v/v acetonitrile was added to a second elution plate. After digestion, the digestion plate and second elution plate were returned to the KingFisher, and the program was resumed to briefly mix and transfer beads to the second elution plate. Both plates were removed, and beads in the second elution plate were separated on a MagnaBot FLEX 96 magnetic plate (Promega). Finally, the supernatants from the digestion plate and second elution plate were combined.

For multiplexed quantification, ~ 100 μg of eluted peptides were labeled with tandem mass tags (TMTpro 18-plex, Thermo Scientific) in a 2.5:1 mass ratio of label:peptides. Samples from the 24 h timepoint were split into technical replicates and included in both TMT plexes to improve batch correction and reproducibility. Briefly, peptides and TMT labels were incubated at 25 °C for 1 h with 600 rpm shaking. Samples were quenched with 5% hydroxylamine at 25 °C for 30 min with 600 rpm shaking. Afterwards, the samples were pooled, desalted using C18 SPE (Sep-Pak, 50 mg columns), and dried in a SpeedVac. Note that TMT labeling schemes include skipped (“empty”) TMT channels (Additional File 3) because total thiol (− NEM) channels yield more cysteine-containing peptides after RAC enrichment (see following subsection). Thus, these "empty" TMT channels were included to prevent isotope intensities from bleeding over to the lower intensity thiol oxidation (+ NEM) channels.

### Resin-assisted capture (RAC) for enrichment of cysteine-containing peptides

Peptide-level resin-assisted capture of cysteine-containing peptides was conducted similar to Day et al. (2022) [200]; however, commercially available agarose beads functionalized with thiol reactive acyl groups (S3^TM^ acyl-RAC capture beads; NANOCS) were used. 200 μg of labeled peptides from each TMT plex were reduced by adding 5 mM DTT and incubating at 37 °C for 30 min. During incubation, 100 μL of S3 acyl-RAC beads were transferred to spin columns (Pierce) attached to a vacuum manifold. The beads were washed with 5 × 500 μL ultrapure water and 5 × 500 μL 25 mM HEPES (pH 7.7). After reduction, 250 mM HEPES (pH 7.7) was added to bring the DTT concentration down to 0.8 mM. Each TMT plex was split such that 100 μg of labeled peptides were incubated with S3 acyl-RAC beads for 2.5 h at RT and 850 rpm shaking.

Following incubation, samples were centrifuged at 1,500 rpm for 1 min, and the flowthrough was collected and stored at − 80 °C for serial enrichment of phosphopeptides. The S3 acyl-RAC beads were extensively washed on the vacuum manifold using 5 × 500 μL of the following in order: 25 mM HEPES (pH 7.0), 2 M NaCl, 80% v/v acetonitrile with 0.1% v/v TFA, and 25 mM HEPES (pH 7.7). To elute cysteine-containing peptides from the S3 acyl-RAC beads, 100 µL of fresh 100 mM ammonium bicarbonate (pH 8.0) containing 20 mM DTT was added to each of the plugged columns. These were then incubated at RT with 1,000 rpm shaking for 30 min. Eluted peptides for the respective TMT plexes were collected in microcentrifuge tubes following 1,500 rpm centrifugation for 1 min. This step was repeated and followed by a final elution using 100 µL of 80% acetonitrile with 0.1% TFA with a 10 min incubation. Finally, samples (including the RAC flowthrough) were dried in a SpeedVac, resuspended in 5% acetonitrile with 0.1% TFA, and desalted using C18 SPE (UltraMicroSpin Columns, The Nest Group). Samples were reconstituted to 0.1 µg/µL in ultrapure water containing 20 mM DTT and 0.01% DDM for LC–MS/MS.

### Immobilized metal affinity chromatography (IMAC) for phosphopeptide enrichment

Phosphopeptide enrichment was performed (without fractionation) as previously described [201] using the remainder of the TMT labeled peptides from SP3 cleanup as well as the peptides in the RAC flowthrough (see previous subsection). Briefly, Fe^3+^ –NTA agarose beads were freshly prepared using the Ni–NTA Superflow agarose beads (QIAGEN). Peptides were resuspended to 0.5 μg/μL in binding/wash buffer containing 80% v/v acetonitrile and 0.1% v/v formic acid. Samples were incubated with 10 μL of Fe^3+^–NTA beads for 30 min at RT and 1,000 rpm shaking. Afterwards, the beads were transferred to in-house prepared stage tips packed with two Empore C18 discs (Supelco). The beads were washed with 2 × 50 μL of wash buffer and once with 50 μL of 1% v/v formic acid. Phosphopeptides were eluted from the beads onto the C18 material using 3 × 70 μL of 500 mM potassium phosphate (pH 7.0). The stage tip was washed with 2 × 100 μL of 1% formic acid, and peptides were eluted into Waters vials containing 0.01% DDM using 2 × 60 μL of 50% acetonitrile with 0.1% formic acid. Samples were dried in a SpeedVac and later reconstituted with 12 μL of 5% acetonitrile containing 0.1% formic acid for LC–MS/MS analysis.

### LC–MS/MS for proteomics samples

Peptides were analyzed on a Vanquish Neo UHPLC coupled with Orbitrap Exploris^™^ 480. Peptides (200 ng for global and 500 ng for redox samples) were separated on a self-packed reverse-phase column (25 cm × 75 μm ID packed with Waters 1.7 μm BEH C18 material) with an integrated PicoTip emitter (New Objective). Each of the redox and phosphopeptide samples were ran twice with 5 μL directly injected onto the column (i.e., no trapping). The column temperature was set at 45 °C with a gradient delivered at 200 nL/min. A binary mobile phase comprised of water with 0.1% formic acid and acetonitrile with 0.1% formic acid was used. 3 h LC gradients were used for global and redox proteomics samples, whereas 2 h was used for phosphoproteomics samples. Data-dependent acquisition was used with the following settings: full MS scans (m/z 300–1800) were collected at a resolution of 60,000 with maximum ion injection time set to automatic mode and automatic gain control set to standard. Fragment ion spectra were collected for up to 20 of the most abundant precursor ions. Precursor ions were first selected (isolation window of 0.7 m/z) and fragmented by higher collisional energy-induced dissociation at 30% normalized collisional energy. MS/MS scans were acquired at a resolution of 30,000 with maximum ion injection time of 200 ms and a normalized automatic gain control of 250%. Fixed first mass was set to 110 m/z to include TMT reporter ions, and dynamic exclusion was 45 s.

### Proteomics data processing and analysis

LC–MS/MS data were searched using MS–GF + [202] against the JGI FASTA file for* Rhodosporidium toruloides* IFO0880 v4.0 [185, 203]. A parent ion mass tolerance of 20 ppm and partial tryptic rule with up to 2 missed cleavages were the key searching parameters. Oxidation on methionine (+ 15.9949) and NEM blocking of cysteine (+ 125.047679) were selected as dynamic modifications. Phosphorylation of serine, threonine, and tyrosine residues (+ 79.9663) was also set as a dynamic modification for the phosphoproteomics searches. TMT labels on peptide N-termini and lysine (+ 304.207146) were selected as fixed modifications. TMT reporter ion intensities were extracted by MASIC [204]. The RStudio package PlexedPiper [205] was used to merge MS–GF + and MASIC data for post-processing of TMT reporter ions and isobaric quantification [206]. These were then processed using a custom pipeline for relative quantification of protein abundance, cysteine thiol oxidation (“Redox”) and phosphorylation (“Phospho”). Refer to the Code availability section for more details.

First, global protein abundance data was acquired by summing up raw TMT reporter ion intensities for corresponding peptides that passed 1% false discovery rate (FDR) filtering. These data were then log_2_-transformed, batch-corrected to account for multiple plexes, and median-centered for normalization. Second, site level PTM data was acquired by aggregating TMT reporter ion intensities for peptides that passed 1% FDR filtering. The log_2_-transformed global peptide data (not batch-corrected) was used to scale the site level PTM abundances to account for TMT channel loadings. Channel-to-channel median centered normalization was then performed, after which these PTM data were batch-corrected. Finally, the average normalized protein abundances (per condition) were subtracted from the corresponding PTM site level abundances. Refer to Additional File 3 for more details. Prior to subtracting global protein abundances, cysteine-containing peptide intensities were used to calculate mean % thiol oxidation. This was accomplished by averaging the normalized raw intensities of thiol oxidation channels on a per condition basis. TMT channels corresponding to pooled total thiol samples (duplicates) were averaged separately. For each site, average thiol oxidation intensities were then divided by the corresponding average total thiol intensities to acquire mean cysteine thiol oxidation %’s.

The RStudio packages limma [195] and MSnSet.utils [https://github.com/PNNL-Comp-Mass-Spec/MSnSet.utils] were used for two-sample *t*-tests, PCAs, and correlation heatmaps. KEGG [207] pathway over-representation analyses were performed using the clusterProfiler package [208]. IDs from pairwise limma *t*-test results with adjusted *p*-values ≤ 0.05 and absolute log_2 _fold changes ≥ 1 were used for KEGG pathway over-representation analysis (Additional File 1, Fig. S7). Enrichment results were reported using cutoffs of *q*-value ≤ 0.2 and adjusted *p*-value ≤ 0.05. For enrichment analysis of reversibly oxidized cysteine sites that correlated with the TG and DG lipidomics data, a complex design was used (~ Nitrogen + Time + Nitrogen:Time) for differential expression analysis. Cysteine sites with Pearson correlation coefficients > 0.80 against TG and DGs were collected, and those with absolute log_2_FCs ≥ 0.8 were used for pathway enrichment analysis. All other figures were made with the “ggplot2” package [198]. Unless specified all log_2_FCs are reported for time-matched comparisons. For example, a log_2_FC of a Cys site at 24 h corresponds to the Low Nitrogen 24 h vs. High Nitrogen 24 h differential expression results. Where available, common gene names are provided based on JGI [81] and eggNOG [82] annotations as well as BLASTp alignment results against* R. toruloides* NP-11 and* S. cerevisiae* S288C [83, 87, 209]. Otherwise, JGI protein accession IDs are provided in the format “RT_####”. Gene nomenclature and their corresponding JGI accession IDs for select annotations are provided in Additional Files 5–7.

### Statistics and reproducibility

All materials used in this study are commercially available, and all procedures used herein are delineated in published protocols—deviations from which are explicitly described in the previous subsections and Additional File 1, Supplementary Methods. The number of replicates in each analysis is provided in the corresponding figure legends. Furthermore, the TMT plex designs are presented in Additional File 3. Database searching parameters and filtering thresholds for tandem mass spectra are detailed in the previous subsection. FDR was estimated using the target-decoy approach [210, 211]. Quantified peptides identified in at least two biological replicates were used for statistical analyses. All *p*-values within each data set were adjusted for multiple comparisons using the Benjamini–Hochberg procedure. Cutoffs used to filter results for enrichment analyses are provided in the respective methods subsections.

## Supplementary Information

Additional file 1. Supporting figures, methods, and materials

Additional file 2. Supplementary lipidomics data including experimental design, raw LC–MS intensities, processed inputs used for analysis using the lipidr package, and results of differential abundance analyses

Additional file 3. Supplementary redox, phospho, and global proteomics data merged with eggNOG annotations for bioinformatics analysis; also includes TMT plex designs

Additional file 4. Supplementary data for percent cysteine thiol oxidation estimates and details regarding calculations

Additional file 5. Select annotations for enzymes involved in nitrogen metabolism and autophagy as well as corresponding differential expression data (global and PTMs) and KEGG representative pathways

Additional file 6. Select annotations for enzymes involved in carbon metabolism, lipogenesis, and antioxidant defense (ROS metabolism) as well as corresponding differential expression data (global and PTMs) and KEGG representative pathways

Additional file 7. Select annotations for proteins involved in TOR, AMPK, calcium, MAPK, PI3K–Akt, and cAMP signaling pathways as well as corresponding differential expression data (global and PTMs) and KEGG representative pathways

## Data Availability

Primary LC–MS raw measurement proteome data (redox, phospho, and global) are openly accessible for download at the Mass Spectrometry Interactive Virtual Environment (MassIVE) community repository under the accession URI https://identifiers.org/massive:MSV000095798 and can be formally cited under https://doi.org/10.25345/C5ZW1943H. Primary LC–MS raw measurement lipidome data (positive and negative ion mode) are openly accessible for download at MassIVE under the accession URI https://identifiers.org/massive:MSV000096720 and can be formally cited under https://doi.org/10.25345/C5K35MS0G. Processed secondary datasets are openly accessible for download and reuse at the PNNL DataHub Predictive Phenomics Initiative (PPI) Project dataset catalog under https://doi.org/10.25584/2510803. Processed proteome data downloads include sample naming keys, raw abundance files, sequence annotations, and supporting metadata materials. Custom proteomics processing source code, *R. toruloides* Nitrogen Limitation Multi-PTM Profiling R Package, is openly accessible from Zenodo and can be formally cited under https://doi.org/10.5281/zenodo.14714224. All other data generated or analyzed during this study are included in this published article and its additional information files.

## Abbreviations

AMPK: AMP kinase
CDW: Cell dry weight
Cer: Ceramide
CL: Cardiolipin
CoQ9: Coenzyme Q9
DG: Diacylglyceride
ER: Endoplasmic reticulum
FA: Fatty acid
FAHFA: Fatty acide ester of hydroxy fatty acids
GC: Glycerophospholipid
GL: Glycerolipid
log_2_FC: Foldchange of log_2_-transformed LC–MS/MS intensities
LPC: Lysophosphatidylcholine
LSEA: Lipid species enrichment analysis
MAPK: Mitogen-activated protein kinase
NEM: N-Ethylmaleimide
OPLS–DA: Orthogonal partial least squares–discriminant analysis
PA: Phosphatidic acid
PC: Phosphatidylcholine
PCA: Principal component analysis
PE: Phosphatidylethanolamine
PG: Phosphatidylglycerol
PI: Phosphatidylinositol
PPP: Pentose phosphate pathway
PR: Prenol lipid
PS: Phosphatidylserine
PTM: Post-translational modification
Redox PTMs: Forms of cysteine thiol oxidation
ROS: Reactive oxygen species
SL: Sphingolipid
SP3: Single-pot, solid-phase-enhanced sample preparation for proteomics
TG: Triacylglyceride
TMT: Tandem mass tag
TOR(C: Target of rapamycin (complex)
