# Supplementary material for: Nitrogen limitation causes a seismic shift in redox state and phosphorylation of proteins implicated in carbon flux and lipidome remodeling in Rhodotorula toruloides
Source: Biotechnol Biofuels Bioprod. 2025 Jul 21;18:80. doi: 10.1186/s13068-025-02657-y (PMC12278674; doi:10.1186/s13068-025-02657-y)
Supplement: Supplementary file 1 — Additional file 1. Supporting figures, methods, and materials [file 13068_2025_2657_MOESM1_ESM.docx]

**Supporting Information:**

Nitrogen Limitation Causes a Seismic Shift in the Redox States and Phosphorylation of Proteins Implicated in Carbon Flux and Lipidome Remodeling for *Rhodotorula toruloides*

Austin Gluth ^a,b^, Jeffrey J. Czajka ^c,d^, Xiaolu Li ^a^, Kent J. Bloodsworth ^a^, Josie G. Eder ^a^, Jennifer E. Kyle ^a^, Rosalie K. Chu ^a^, Bin Yang ^b^, Wei-Jun Qian ^a^, Pavlo Bohutskyi ^a,b^, Tong Zhang ^a,*^

1. Biological Sciences Division, Pacific Northwest National Laboratory, Richland, WA, USA
2. Department of Biological Systems Engineering, Washington State University, Richland, WA, USA
3. Energy and Environment Directorate, Pacific Northwest National Laboratory, Richland, WA, United States
4. Agile BioFoundry, US Department of Energy, Emeryville, CA, United States

* Corresponding Author

Dr. Tong Zhang

Biological Sciences Division

Pacific Northwest National Laboratory

Richland, WA 99352

**Tel:** (509)371-7780

**Email:** [tong.zhang@pnnl.gov](mailto:tong.zhang@pnnl.gov)

**Contents:**

(A) Supplementary Figures (Pgs. S3–17)

(B) Supplementary Tables (S18)

(C) Methods (S19)

(D) References (S20)

**A. Supplementary Figures**

**
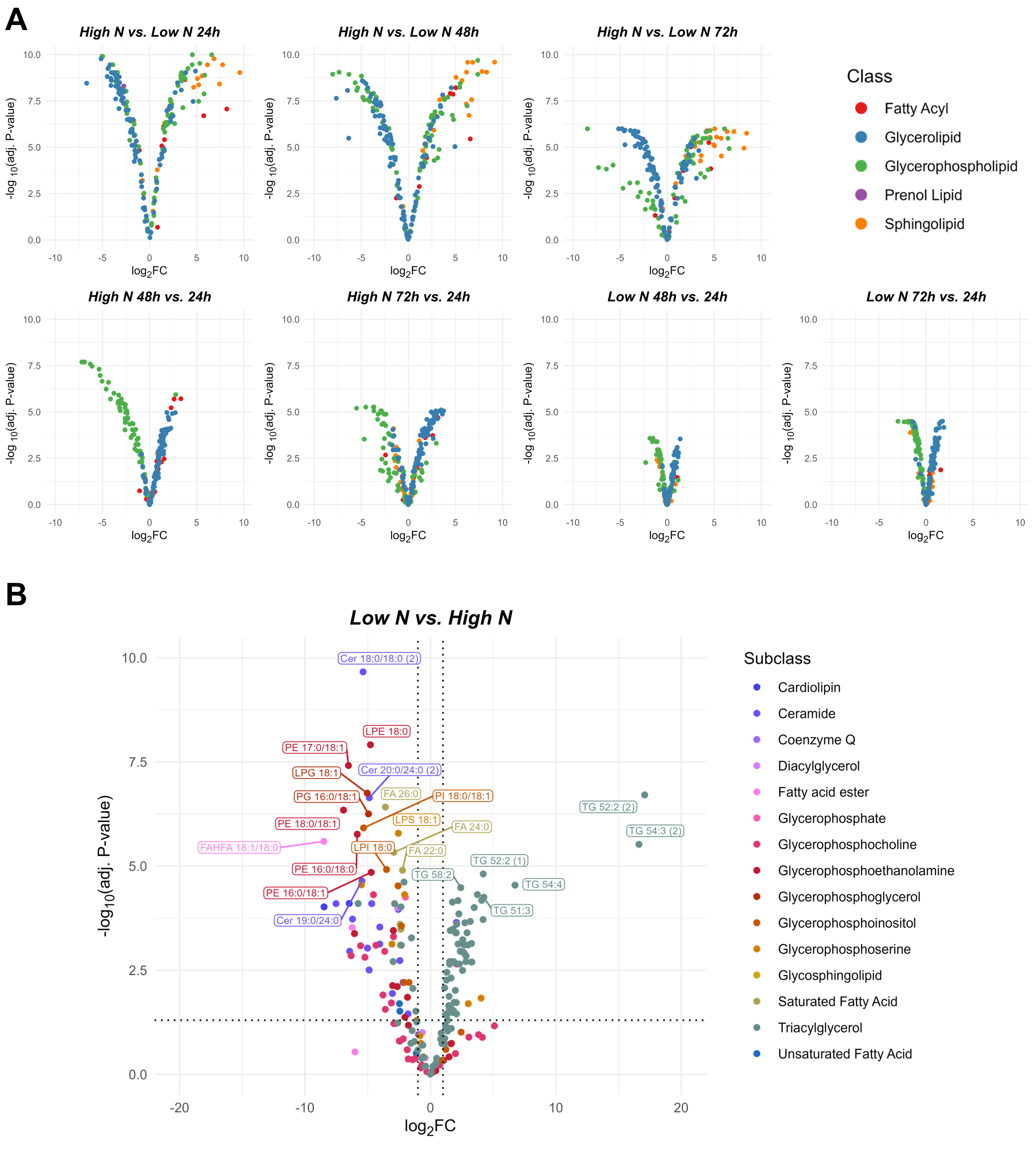
Supplementary Fig. 1.** Differential expression analyses discriminate lipids according to nitrogen availability. ***A*,** Volcano plots of differential expression results for select comparisons. ***B*,** Volcano plot of differential expression results modeling (~ Nitrogen + Time + Nitrogen:Time) the effect of nitrogen and time on lipid abundances. See the legend in the adjacent panel for lipid subclass color codes. Abbreviations are as follows: ceramide (Cer), cardiolipin (CL), prenol lipid (PR), triacylglyceride (TG), diacylglyceride (DG), fatty acid (FA), fatty acid ester (FAHFA), glycosphingolipid (HexCer), phosphatidylcholine (PC), phosphatidylethanolamine (PE), phosphatidylglycerol (PG), phosphatidylinositol (PI), phosphatidylserine (PS), phosphatidic acid (PA), and the corresponding lyso- derivatives (e.g., LPC).

**
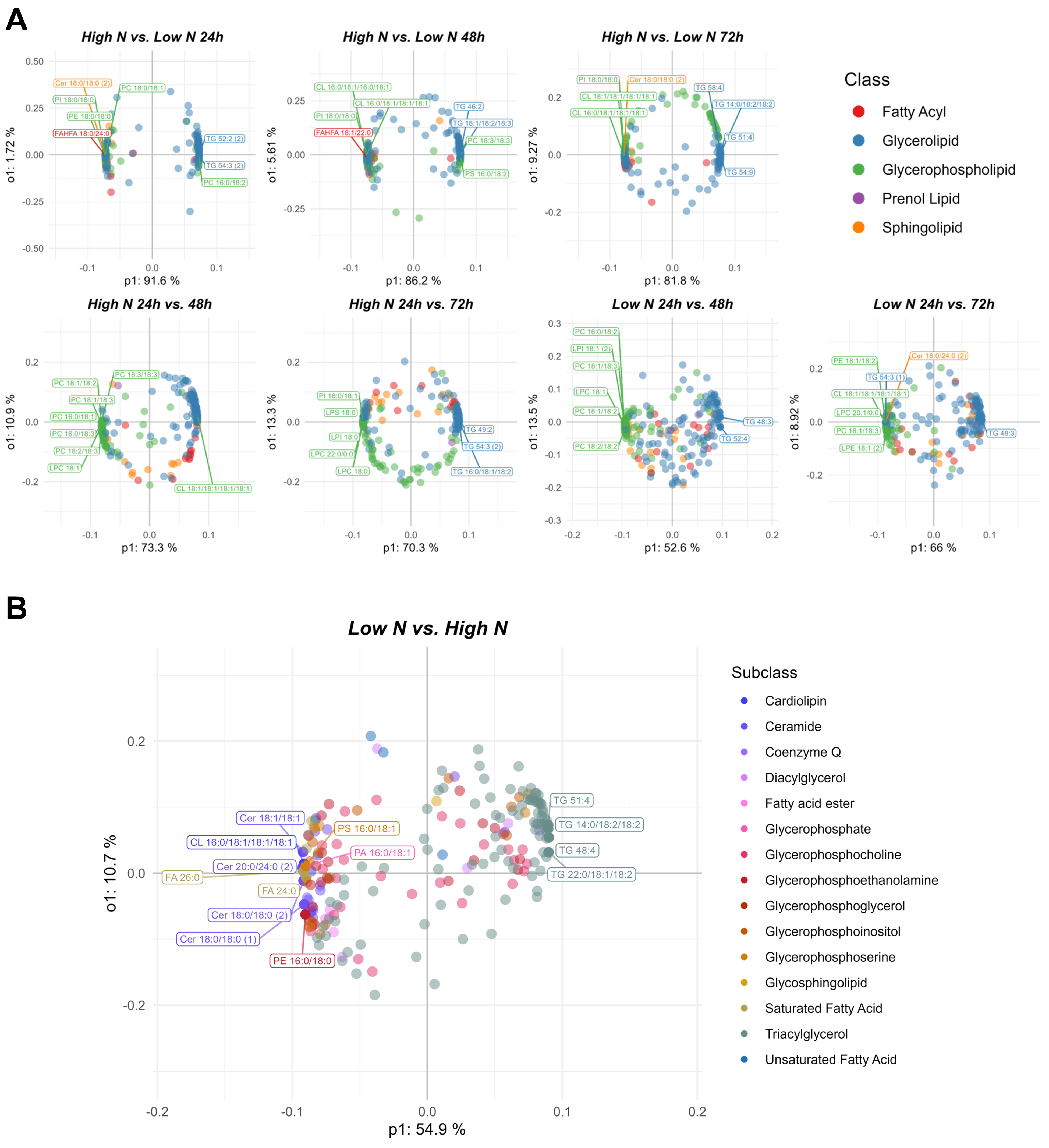
 Supplementary Fig. 2.** Supervised multivariate analyses rank lipids according to nitrogen availability and time. ***A*,** Orthogonal partial least squares discriminant analysis (OPLS-DA) results for specific sample type comparisons (e.g., high nitrogen 24 h vs. low nitrogen 24 h). ***B*,** OPLS-DA was also performed using nitrogen as a discrete grouping variable (high vs. low) to evaluate lipidome differences. The top 14 lipids are labeled in each plot according to highest separation of the predictive component (“p1” x-axis label) and lowest intragroup variability (orthogonal “o1” y-axis label). This allows one to model the effect of nitrogen (p1) vs. other factors like time or micronutrient availability that contribute to intragroup variability (o1). See the legend in the adjacent panel for lipid subclass color codes.


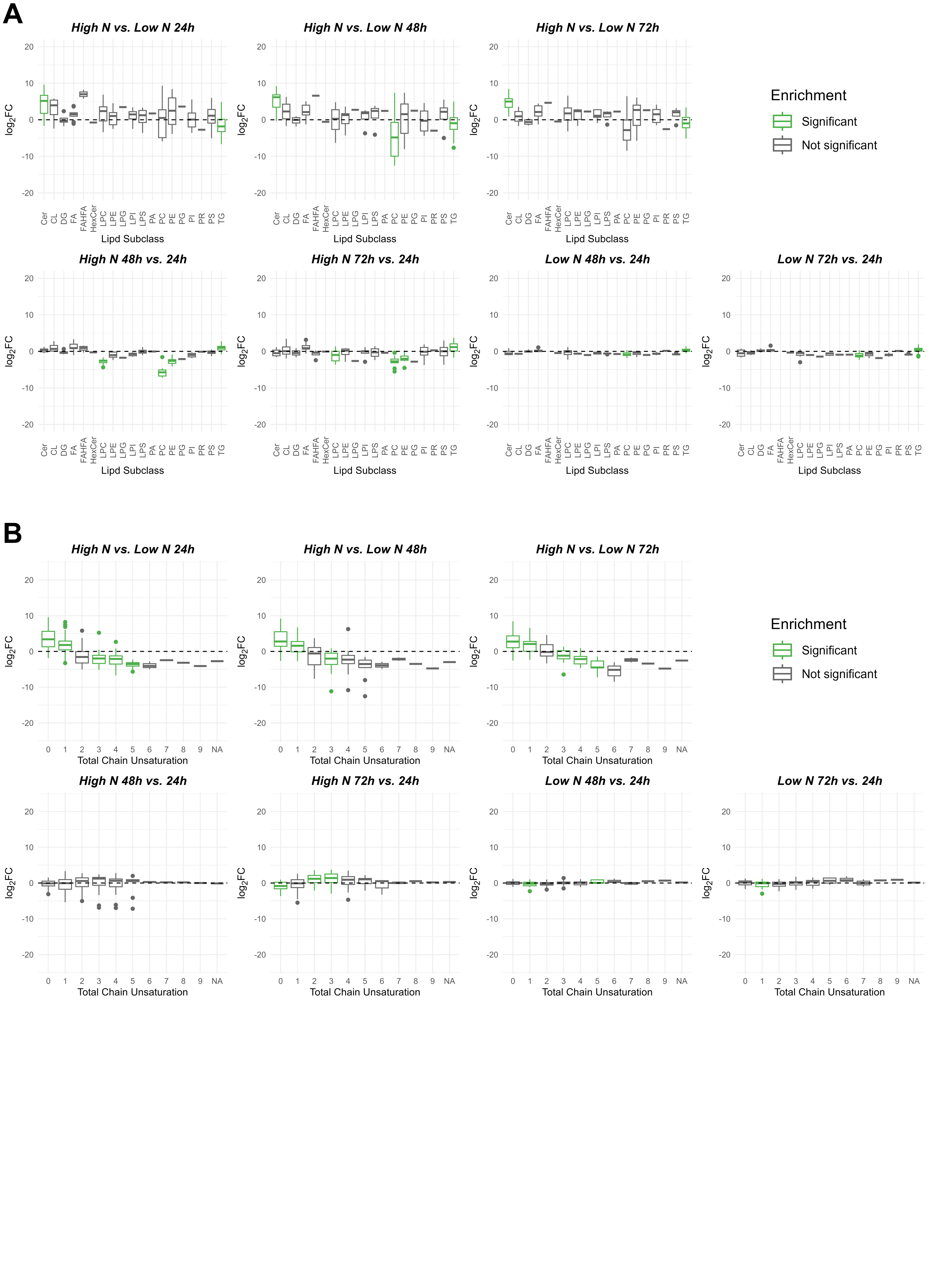


**Supplementary Fig. 3.** Lipid species enrichment analyzes (LSEA) highlight nitrogen-dependent changes in lipid subclasses and degrees of unsaturation. ***A*,** LSEA results for lipid subclasses for selected comparisons. ***B*,** LSEA results for total chain unsaturation: note that, for example, degrees of unsaturation for a TG would be summed for all three acyl groups. “NA” corresponds to the prenol lipid coenzyme Q9. Refer to the legend of Supplementary Fig. 1 for abbreviation definitions.


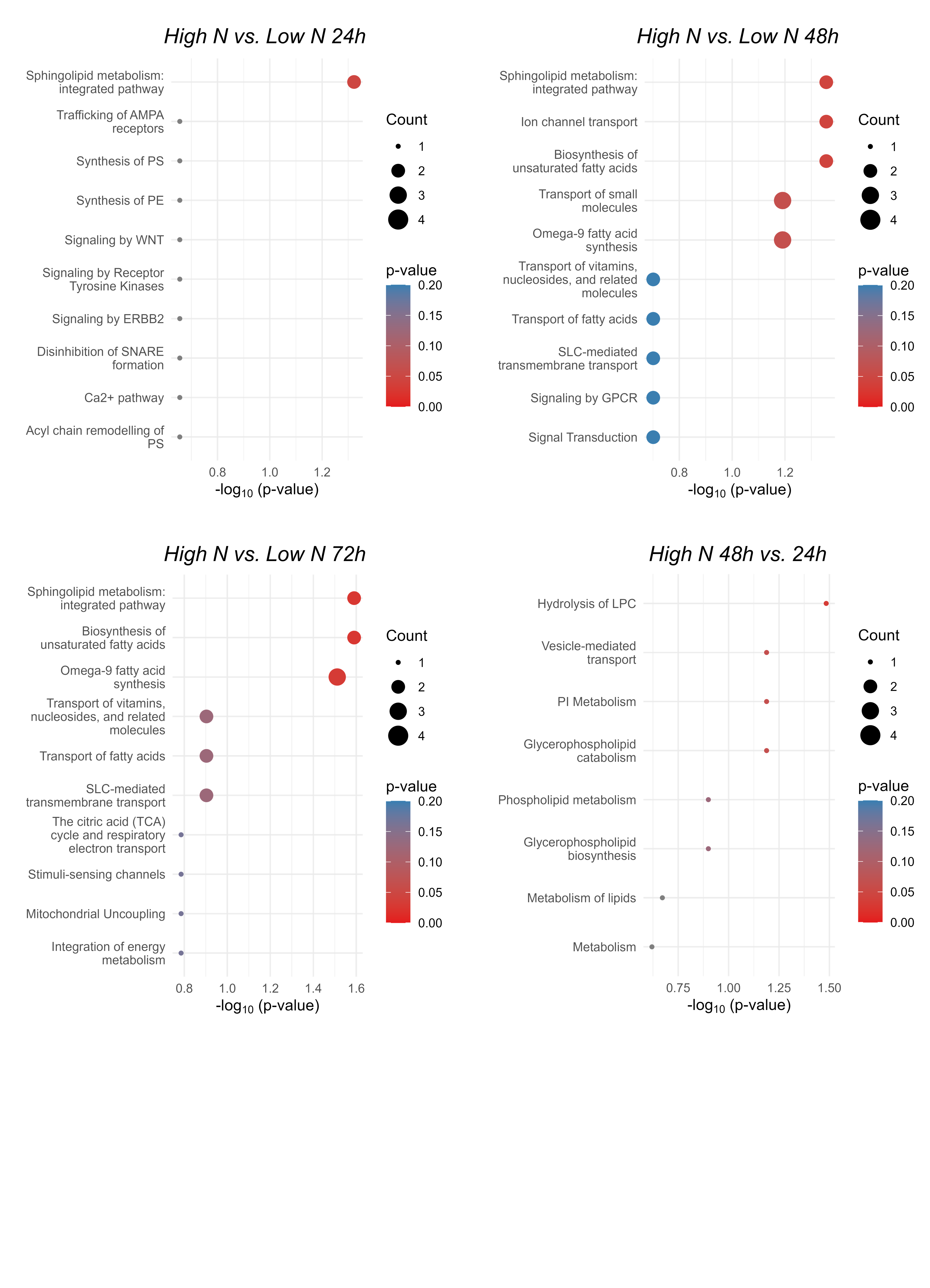
**Supplementary Fig. 4.** Pathway enrichment of lipidomics data detailing expected and unexplored pathways relating to nitrogen limitation in oleaginous yeast. Over-representation analysis of enriched metabolite sets (MSEA-ORA) was performed for all comparisons; however, only those shown here resulted in significant enrichment results. An absolute log_2_FC cutoff of 4 with an adjusted p-value ≤ 0.05 was used to filter differential expression results used for MSEA-ORA.


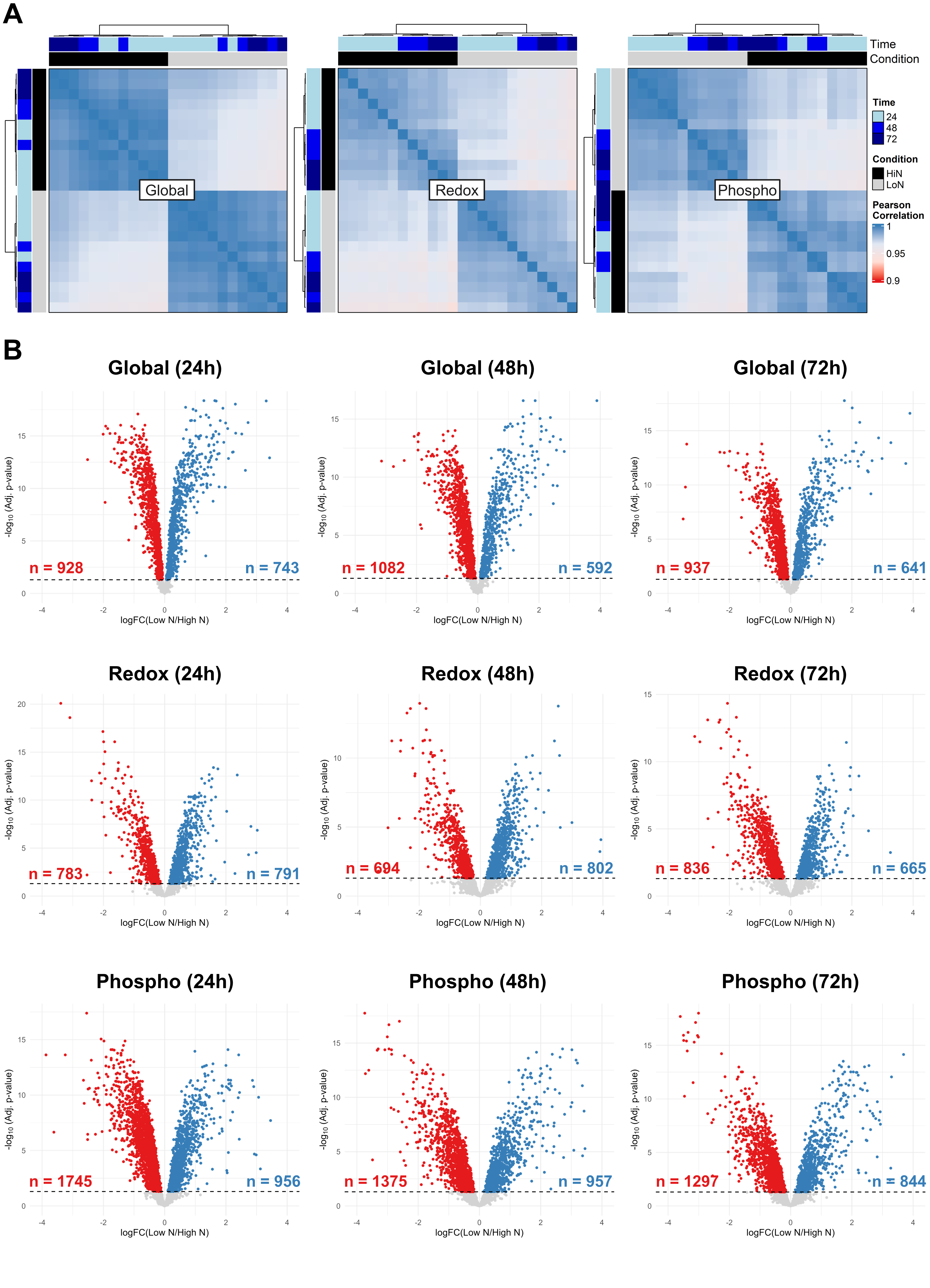


**Supplementary Fig. 5.** Nitrogen availability and cultivation time affect protein abundances and PTMs. ***A,*** Clustered heatmaps presenting Pearson correlations among high and low nitrogen conditions over three time points. ***B,*** The volcano plots contain –log_10_ limma adjusted p-values on the y-axis and log_2_ transformed fold changes (Low Nitrogen/High Nitrogen) on the x-axis. The dotted line in each plot specifies an adjusted p-value of 0.05. Unique protein and site IDs are included in each plot along with the number of IDs that passed the adjusted p-value threshold and were upregulated (“Up”) or downregulated (“Down”).


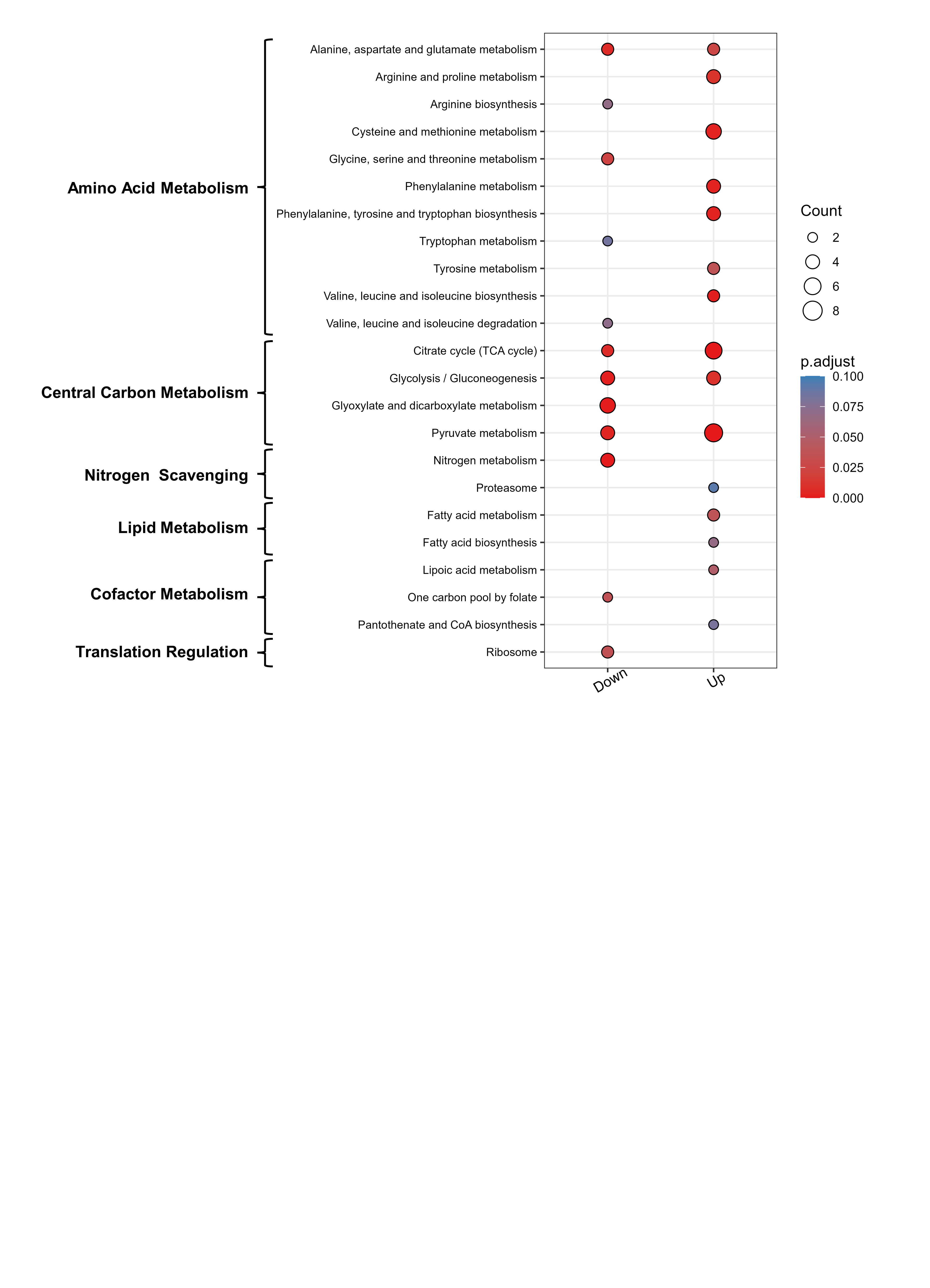


**Supplementary Fig. 6.** Over-representation analysis of enriched KEGG pathways using a subset of redox proteomics data that correlated with TG and DG abundance trends. Cysteine sites with Pearson correlation coefficients > 0.80 were first collected. Those with absolute log_2_FCs ≥ 0.8 from differential expression analysis using a limma linear model (~ Nitrogen + Time + Nitrogen:Time) were filtered for KEGG pathway enrichment analysis.


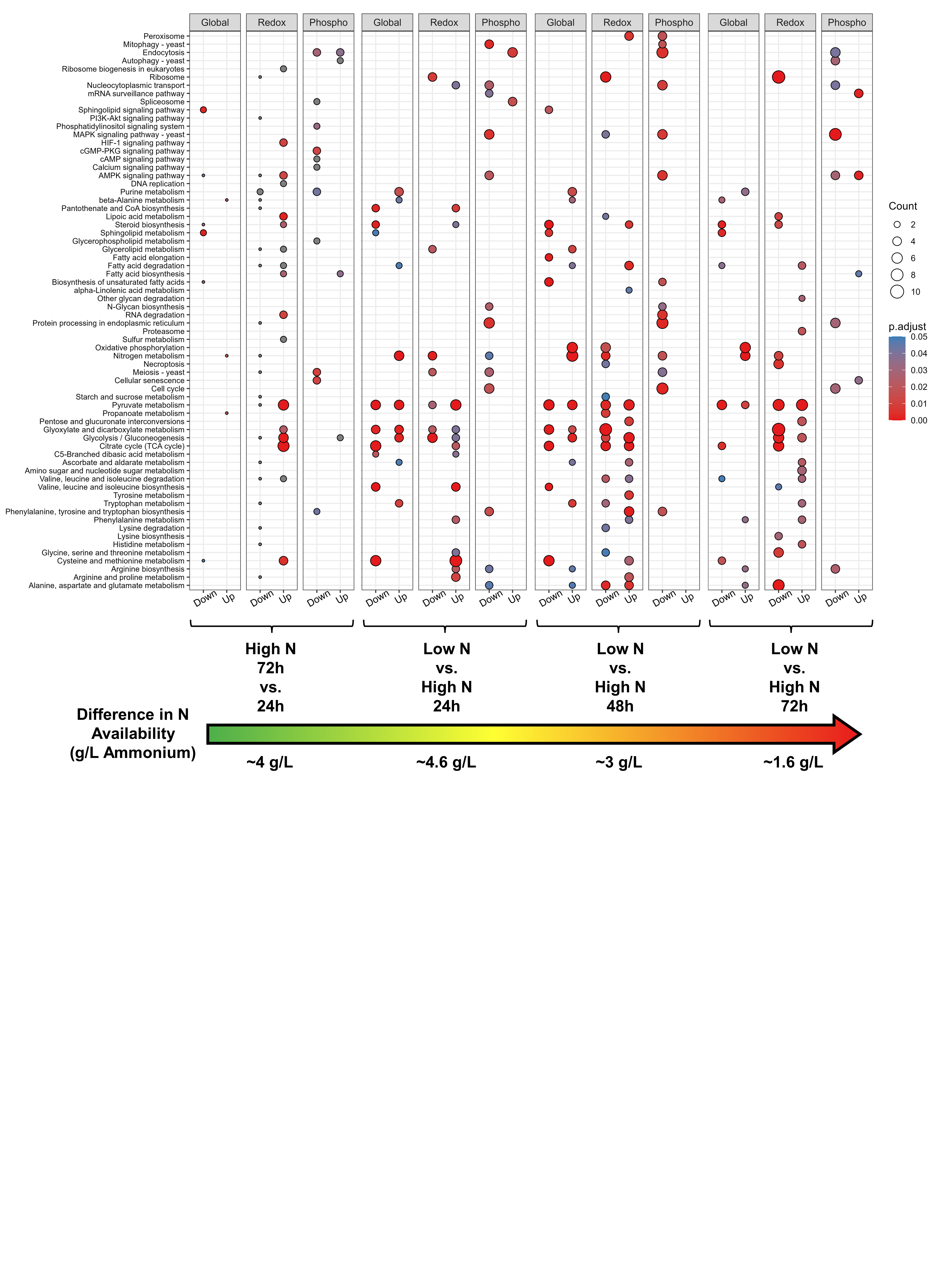


**Supplementary Fig. 7.** Over-representation analysis of enriched KEGG pathways using pairwise differential expression analyses. IDs from pairwise limma t-test with adjusted p-values ≤ 0.05 and absolute fold changes ≥ 1 were used for KEGG enrichment.


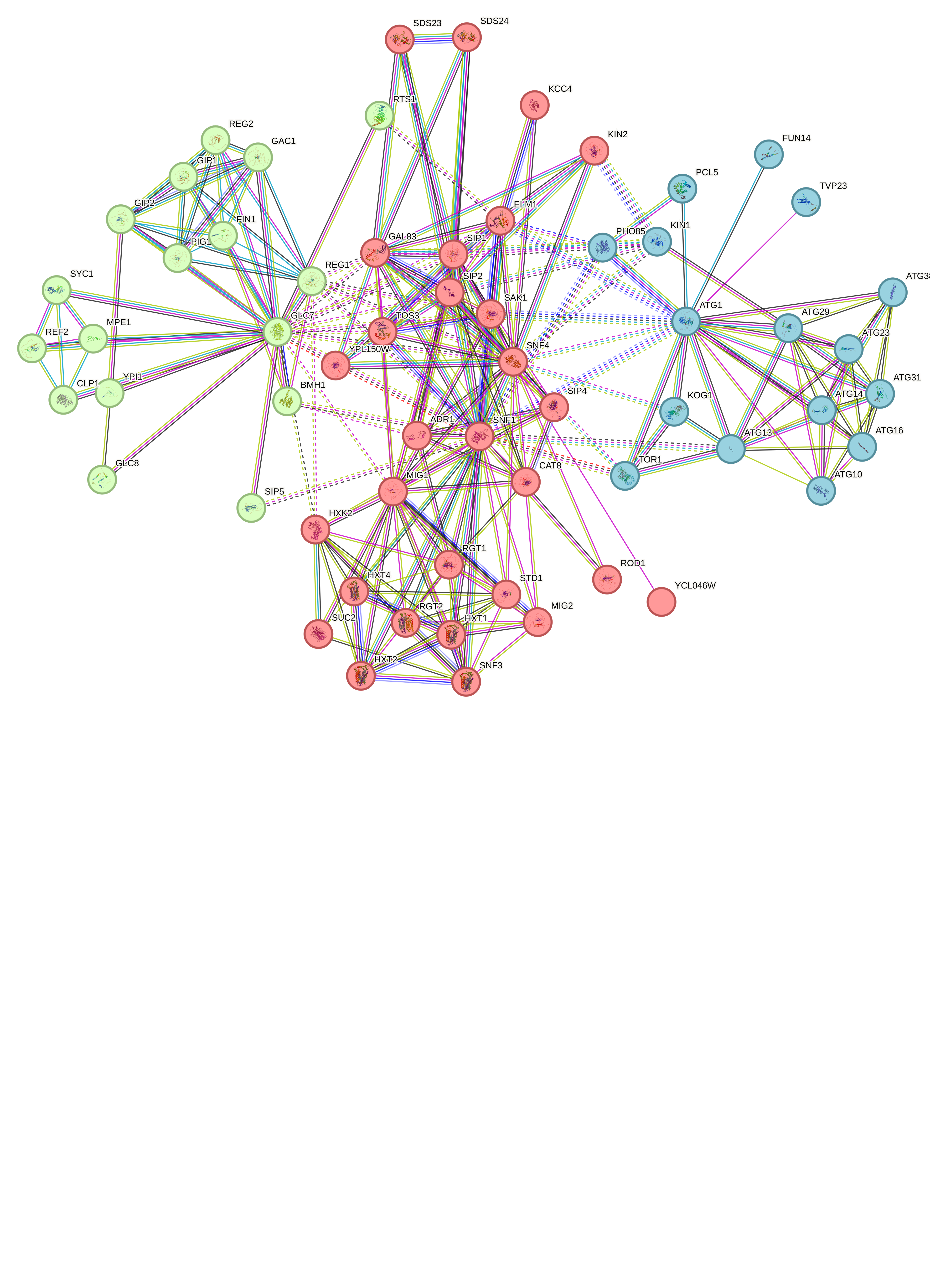
**Supplementary Fig. 8.** A STRING network [1] of functional and physical protein associations using *S. cerevisiae* SAK1 (serine/threonine kinase upstream of SNF1) as an input. These results provide a starting point for exploring signaling pathway interplay in *R. toruloides*. Many of the proteins (nodes) shown here were manually annotated in *R. toruloides* and are included in **Supplementary Data 6**. However, we acknowledge that there are likely important differences between these yeasts. Data were organized according to k-means clustering with three clusters. A minimum interaction score of 0.700 was used, and network edges designate the type of interaction evidence. Please refer to Szklarczyk et al. [1] and http://string-db.org/ for more information.


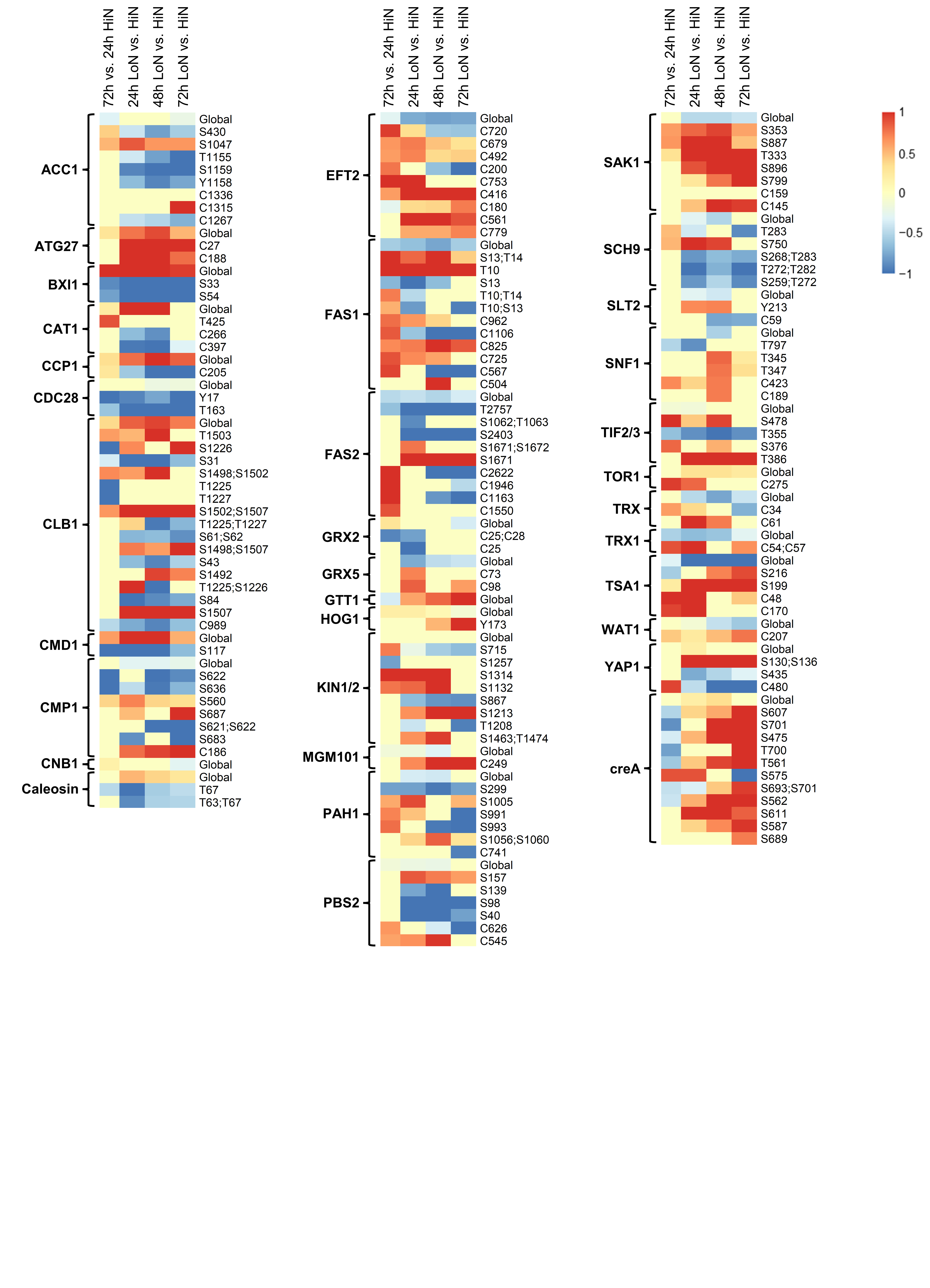
**Supplementary Fig. 9.** Heatmaps of differential expression results for select antioxidants and signaling pathway enzymes. These log_2_FC results correspond to those summarized in **Fig. 5** of the main text, and a gradient color legend is provided in the top right. Insignificant differences (adj. p-value > 0.05 or log_2_FC = 0) in protein or PTM abundances are shown in yellow. For JGI protein IDs, refer to **Supplementary Data 6**.


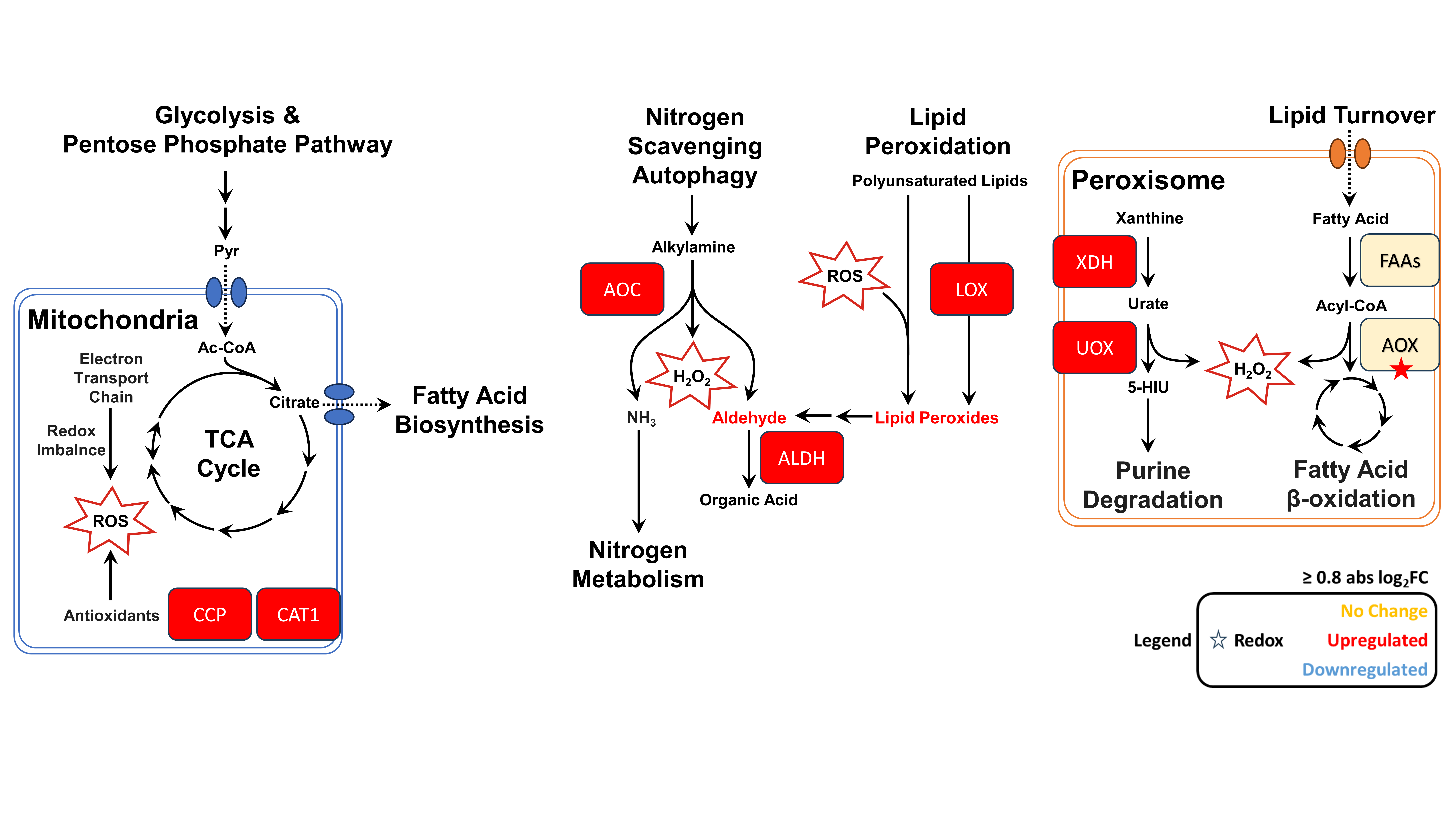
**Supplementary Fig. 10.** Overview of upregulated ROS-generating processes observed during nitrogen limitation. Results for enzymes with absolute log_2_ foldchanges ≥ 0.8 are summarized. Enzyme abbreviations are as follows: CCP, Cytochrome c Peroxidase; CAT1, Catalase; AOC, Copper Amine Oxidase; ALDH, Aldehyde Dehydrogenase; LOX, Linoleate 8R-lipoxygenase; XDH, Xanthine Dehydrogenase; UOX, Urate Oxidase; FAA, Fatty Acid-CoA Ligase; AOX, Acyl-CoA Oxidase.

**B. Supplementary Tables**

**Supplementary Table 1.** Summary of rowth, substrate utilization, and lipid production kinetics.

| Initial C:N | *μ* (h^-1^)^**^ | *X*_72h_ (g/L) | *Y_X/S_* | *P*_72h_ (g/L) | *Y_P/S_* | *r_S_* (g/L/h) | *r_P_* (g/L/h) | *q_S_* | *q_P_* |
| --- | --- | --- | --- | --- | --- | --- | --- | --- | --- |
| 5:1 | 0.130 | 9.56 | 0.638 | 0.97 | 0.065 | 0.208 | 0.014 | 0.022 | 0.001 |
| 90:1 | 0.120 | 8.27 | 0.565 | 2.27 | 0.155 | 0.203 | 0.032 | 0.025 | 0.004 |

Averages are reported for the following: *X*_72h_ (total biomass titer at 72 h), *Y_X/S_* (total biomass yield from glucose), *P*_72h_ (lipids titer at 72 h), *Y_P/S_* (lipids yield from glucose), *r_S_* (rate of glucose consumption over 72 h), *r_P_* (rate of lipid production over 72 h), *q_S_* (specific glucose uptake rate over 72 h; g consumed glucose / g biomass / h), *q_P_* (specific lipid production rate over 72h; g lipids / g biomass / h).

^**^ Growth rates (μ) were estimated linearly using CDWs from 0 h and 24 h. According to analysis using the Growthcurver package [2], log-phase in the low nitrogen condition occurs somewhere around the 24 h time point. Values from using the Growthcurver package are 0.062 and 0.106 for the high and low nitrogen conditions, respectively. These results should be interpreted carefully within the context of our experimental approach, which employed a relatively high initial cell density inoculum and sampling time points that likely did not capture all growth phases completely. This approach was intended to document key snapshots of nitrogen limitation and starvation effects on yeast cellular metabolism—specifically how alterations in redox state and phosphorylation of regulatory proteins relate to shifts in lipid metabolism—rather than to provide comprehensive growth kinetics.

**C. Supplementary Materials and Methods**

**SP3 Bead Information and Pre-washing.** The following magnetic beads were used for single-pot, solid-phase-enhanced sample preparation (SP3): Sera-Mag SpeedBead Carboxylate-Modified E3 (GE Healthcare, 65152105050250, 50 μg/μL, hydrophobic) and E7 (GE Healthcare, 45152105050250, 50 μg/μL, hydrophilic) [3,4]. The beads were pre-washed following recommendations from Cytiva. After removing from storage at 4 °C, beads are equilibrated to RT for 30 min. Beads were resuspended by gentle mixing, and a 1:1 mix of hydrophobic and hydrophilic beads were prepared (up to 25 mg in a 1.5 mL microcentrifuge tube). This tube was placed on an Invitrogen DynaMag-2 magnetic stand for 2 min, and the storage buffer was aspirated. The beads were washed by adding 1 mL of 50 mM HEPES (pH 9.1) containing 200 mM NaCl. The beads were then incubated at RT and 850 rpm shaking for 2 min and placed again on the magnetic stand for 2 min to collect the beads and remove the wash buffer. This procedure was repeated twice more followed by additional washes using 1 M NaCl to help remove residual bead encapsulation polymer. Finally, beads were washed five times with ultrapure water and resuspended at a final concentration of 50 μg/μL.

**D. References**

1. Szklarczyk D, Kirsch R, Koutrouli M, Nastou K, Mehryary F, Hachilif R, et al. The STRING database in 2023: protein–protein association networks and functional enrichment analyses for any sequenced genome of interest. Nucleic Acids Res. 2023;51:D638–46.

2. Sprouffske K, Wagner A. Growthcurver: an R package for obtaining interpretable metrics from microbial growth curves. BMC Bioinformatics. 2016;17:172.

3. Hughes CS, Foehr S, Garfield DA, Furlong EE, Steinmetz LM, Krijgsveld J. Ultrasensitive proteome analysis using paramagnetic bead technology. Mol Syst Biol. 2014;10:757.

4. Hughes CS, Moggridge S, Müller T, Sorensen PH, Morin GB, Krijgsveld J. Single-pot, solid-phase-enhanced sample preparation for proteomics experiments. Nat Protoc. 2019;14:68–85.
